# MRD as Biomarker for Response to Donor Lymphocyte Infusion after Allogeneic Hematopoietic Cell Transplantation in Patients with AML

**DOI:** 10.3390/cancers15153911

**Published:** 2023-08-01

**Authors:** Katrin Teich, Michael Stadler, Razif Gabdoulline, Jyoti Kandarp, Clara Wienecke, Bennet Heida, Piroska Klement, Konstantin Büttner, Letizia Venturini, Martin Wichmann, Wolfram Puppe, Christian Schultze-Florey, Christian Koenecke, Gernot Beutel, Matthias Eder, Arnold Ganser, Michael Heuser, Felicitas Thol

**Affiliations:** 1 Department of Hematology, Hemostasis, Oncology, and Stem Cell Transplantation, Hannover Medical School, 30625 Hannover, Germanyheuser.michael@mh-hannover.de (M.H.); 2 Department of Virology, Hannover Medical School, 30625 Hannover, Germany

**Keywords:** donor lymphocyte infusions (DLIs), allogeneic hematopoietic cell transplantation (alloHCT), measurable residual disease (MRD), targeted graft-versus-leukemia (GvL) effect

## Abstract

**Simple Summary:**

Donor lymphocyte infusions (DLIs) are immune cells of the donor. They can potentially directly target leukemic cells. Thus, DLIs can be given to acute myeloid leukemia (AML) patients after allogeneic hematopoietic cell transplantation (alloHCT) in order to prevent or treat relapse. Measurable residual disease (MRD) describes very low levels of disease. Currently, it is still not well described how we can use MRD assessment for predicting response and outcome after DLI. This is a retrospective study looking at 76 AML patients receiving DLI treatment. MRD was evaluated prior to DLI treatment as well as 30 and 90 days after DLI. It was observed that within 90 days after DLI treatment, 73% of MRD^+^ patients converted to MRD^−^. Furthermore, MRD and remission status at the time of DLI were highly prognostic for the outcome (both for the relapse rate and also for relapse-free survival).

**Abstract:**

Donor lymphocyte infusions (DLIs) can directly target leukemic cells through a graft-versus-leukemia effect and play a key role in the prevention and management of relapse after allogeneic hematopoietic cell transplantation (alloHCT). Predictors of response to DLIs are not well established. We evaluated measurable residual disease (MRD) before, 30 and 90 days after DLI treatment as biomarkers of response. MRD was assessed by next-generation sequencing in 76 DLI-treated acute myeloid leukemia patients. MRD status before DLI treatment was independently prognostic for event-free survival (EFS, *p* < 0.001) and overall survival (OS, *p* < 0.001). Within 90 days of DLI treatment, 73% of MRD^+^ patients converted to MRD^−^ and 32% of patients without remission achieved remission. MRD status 90 days after DLI treatment was independently prognostic for the cumulative incidence of relapse (CIR, *p* = 0.011) and relapse-free survival (RFS, *p* = 0.001), but not for OS. To evaluate the role of DLI treatment in MRD^−^ patients, 23 MRD^−^ patients who received DLIs were compared with a control cohort of 68 MRD^−^ patients not receiving DLIs. RFS (*p* = 0.23) and OS (*p* = 0.48) were similar between the two cohorts. In conclusion, MRD is prognostic before (EFS, OS) and after (CIR, RFS) DLI treatment and may help in the selection of patients who benefit most from DLIs.

## 1. Introduction

Relapse of acute myeloid leukemia (AML) or myelodysplastic syndrome (MDS) after allogeneic hematopoietic cell transplantation (alloHCT) remains a major challenge [1,2,3]. Donor lymphocyte infusions (DLIs) after alloHCT can target leukemic cells through the enhancement of the graft-versus-leukemia (GvL) effect, which can reduce the relapse risk or even treat clinical relapse [1,2,4,5,6]. 

However, graft-versus-host disease (GvHD) can limit the beneficial impact of the GvL effect [4,5,7,8,9]. GvHD is especially problematic in the setting of prophylactic or preemptive applications of DLIs, as it might shift the benefit–risk ratio to an unfavorable outcome. Therefore, it is important for a prophylactic DLI treatment to select patients who benefit the most from targeted DLI treatment, specifically focusing on patients with high relapse risk.

While there are clinical parameters that are prognostic for a better response to DLIs [7,10,11,12,13], molecular markers for response are still missing. Additionally, the role of measurable residual disease (MRD) in the setting of DLIs is unclear. While molecular MRD is predictive for the response to chemotherapy and outcome after alloHCT [14,15,16,17], it is unknown whether MRD is helpful to stratify the use of DLIs in patients with a lower-risk disease. NGS-based MRD assessment is a type of molecular MRD that be widely applied. Unlike RT-qPCR which is only applicable to a few abnormalities (e.g., NPM1 and core-binding factor AML), NGS-based MRD is not restricted to specific genetic aberrations [18]. However, we have learned that mutations associated with clonal hematopoiesis, such as *DNMT3A*, *TET2* and *ASXL1* (DTA mutations), should not be used for MRD assessment [18]. 

We aimed to investigate in a retrospective analysis if molecular markers found at diagnosis are predictive of the outcome after DLI, evaluate NGS-based MRD as a prognostic marker before and after DLI and analyze the therapeutic role of DLI in MRD-negative (MRD) patients.

## 2. Materials and Methods

### 2.1. Patients and Treatment

AML and MDS/AML patients according to the 2022 European LeukemiaNet (ELN) recommendations [19] (excluding acute promyelocytic leukemia) were included in this retrospective study if they were aged ≥18 years, received alloHCT and DLI treatment at Hannover Medical School (MHH) between 1998 and 2018 and had peripheral blood (PB) and/or bone marrow (BM) DNA samples available. Patients were part of the AMLSG Biology and outcome study (NCT01252485). Written informed consent was obtained according to the Declaration of Helsinki. The study was approved by the institutional review board of MHH (ethical votes 936/2011 and 3432-2016). The primary cohort consisted of 38 patients receiving preemptive DLIs (pDLIs), meaning that the patient was in complete remission (CR) or CR with incomplete hematologic recovery (CRi) without evidence of relapse. Various reasons led to treatment with DLIs in this cohort (treatment protocol, mixed chimerism, compensation for a T-cell-depleted stem cell boost and high-risk cytogenetics at diagnosis).

Another 38 patients received therapeutic DLIs (tDLIs), which were defined as DLIs to treat clinical relapse after alloHCT, either alone or in combination with chemotherapy or small molecule inhibitors. Importantly, MRD assessment was performed post hoc and did not influence therapeutic decisions.

The control cohort, published by Heuser et al. in 2021 [14], included 68 AML patients (excluding acute promyelocytic leukemia) aged ≥18 years who underwent alloHCT between 2000 and 2017 at MHH and did not receive DLIs after alloHCT. These patients were in CR or CRi after alloHCT. 

### 2.2. Cytogenetic and Molecular Analysis

Peripheral blood or BM samples from diagnosis were studied centrally by G- and R-banding analysis. Chromosomal abnormalities were described according to the International System for Human Cytogenetic Nomenclature [20].

DNA from ficoll-separated BM or PB samples was extracted using the Allprep DNA/RNA purification kit (Qiagen, Hilden, Germany). DNA from whole blood cell pellets was extracted using the DNeasy Blood and Tissue Kit (Qiagen, Hilden, Germany). The peqGOLD Micro Spin Tissue DNA Kit was used to extract DNA from cells taken from BM smears (VWR International bvba, Leuven, Belgium). DNA libraries were prepared from diagnostic DNA samples with a custom TruSight Myeloid Panel (Illumina, San Diego, CA, USA) covering genes or gene hotspots of 46 genes recurrently found in myeloid leukemia as previously described [14,15,21] or with a Custom enrichment panel (Nextera Flex for enrichment, lllumina) covering 48 genes (Supplementary Table S1) according to the manufacturers’ instructions. The Illumina Miseq reagent kit v3 (600 cycles) was used for sequencing and was run on the MiSeq sequencer (Illumina).

### 2.3. Error-Corrected Sequencing of Patient-Specific Mutations

Custom amplicon sequencing for sensitive detection of single-nucleotide variants and insertions/deletions was performed as previously described [15]. Briefly, to reduce the sequencing error rate, we used a proofreading polymerase for PCR, introduced random barcodes to allow bioinformatic error correction, performed the initial PCR with only 5 PCR cycles, avoided identical multiplex identifier (MID)/gene combinations on consecutive MiSeq runs and applied two methods of bioinformatic error correction (see Supplementary Methods). Sequencing was performed on the MiSeq sequencer, aiming for a high coverage per sample, and run forward and reverse with 251 cycles. This method of error-corrected sequencing of patient-specific mutations was performed before (range 0–96 days) DLI, 30 days (FU30) and 90 days (FU90) after the first DLI (Supplemental Figure S1). All mutations detected at diagnosis were used for MRD monitoring. *DNMT3A*, *TET2* and *ASXL1* mutations were excluded as MRD markers according to the ELN MRD working group recommendations [14]. Mutations were categorized into mutational classes for analysis (Supplementary Table S2). 

### 2.4. Bioinformatics and Statistical Analyses

Bioinformatics analysis of myeloid panel results and error-corrected NGS-MRD sequencing was performed as previously described [15,21].

Median follow-up was calculated by the reverse Kaplan–Meier method. Outcome and endpoints were defined as described previously [22] and were calculated with the Kaplan–Meier method. Overall survival (OS) and event-free survival (EFS) were measured from the date of the first DLI. Relapse-free survival (RFS), cumulative incidence of relapse (CIR) and non-relapse mortality (NRM) were calculated from the date of CR/CRi after the first DLI. The Kaplan–Meier method and log-rank test were used to estimate the distribution of OS, EFS and RFS and compare differences between survival curves, respectively. The Gray test was used for CIR and NRM plots, accounting for competing risks [23]. 

Variables were compared using the Kolmogorov–Smirnov test and Kruskal–Wallis test for continuous variables and the Chi-squared test for categorical variables for exploratory purposes.

The two-sided level of significance was set at *p* < 0.05. The statistical analyses were performed with the statistical software package SPSS 27.0 (IBM Corporation, Armonk, NY, USA), statistical program R and RStudio using packages “survival”, “cmprsk”, “aod”, “foreign” and “openxlsx” (R Core Team 2020, R Foundation for Statistical Computing, Vienna, Austria), Microsoft Excel 2016 (Microsoft Corporation, Redmond, WA, USA), GraphPad PRISM Version 9 for Windows (GraphPad Software, San Diego, CA, USA) and custom Linux scripts. 

Categorized variables were considered in univariate analysis for CIR, NRM, RFS and OS for patients in CR/CRi at FU30 or FU90. 

The Wald test was used for OS, EFS and RFS, and the Gray test from the Fine-Gray model was used for CIR and NRM in univariate and multivariate analyses [24]. 

Variables were used for multivariate analysis if *p* ≤ 0.11 in univariate analysis. Variables with >33% missing values were not used for multivariate analysis. For multivariate analysis, a Cox proportional hazards model was constructed for OS, RFS and EFS, adjusting for potential confounding covariates. Variables in multivariate analysis were reduced by backward elimination by removing the variable with the worst fit. The multivariate analysis was selected from this series of models when the *p*-values of all variables were smaller than 0.05.

## 3. Results

### 3.1. Patient and Treatment Characteristics

Of 95 patients, 19 patients were excluded as described in Supplementary Figure S2, resulting in a total cohort of 76 patients. The median age at diagnosis was 52.9 years. Forty-four (58%) patients had de novo AML, while thirty-two (42%) patients had secondary AML (sAML), therapy-related (tAML) AML or MDS/AML (Table 1). According to the 2022 ELN classification, 31 (41%) patients had favorable or intermediate risk, and 45 (59%) patients had adverse risk AML. The median time between alloHCT and DLI was 7.4 months (range 3.1–74.7 months) (Supplementary Table S3). 

Mutations were detected in 41 genes—the most frequently used are *DNMT3A, TP53, FLT3, RUNX1* and *TET2*. At diagnosis, patients showed mutations most frequently in epigenetic modifiers (n = 36, 47%), signal transduction genes (n = 29, 38%) and myeloid transcription factors (n = 27, 36%) (Supplementary Table S4). 

A median of 2 molecular aberrations was used for MRD monitoring per patient (range 1–5) (Supplementary Table S5). In total, 466 MRD analyses were performed on 76 patients (406 in PB and 60 in BM). The median limit of detection (LOD) was 0.022% (range 0–3.683%), and the median VAF of MRD-positive (MRD^+^) markers was 3.355% (range 0.0044–98.58%). The median follow-up time of our cohort was 11.3 years.

### 3.2. Impact of Mutations and Baseline Characteristics at Diagnosis on Outcome of Patients Receiving DLIs

We evaluated whether the mutational profile found at the time of diagnosis had a direct prognostic impact on patients receiving DLIs. Given the small number of patients within the gene groups, we stratified mutations according to mutational classes (see Supplementary Table S2). First, we looked at CR/CRi status at FU90 and found a trend for an association of chromatin-modifier mutations with a higher CR/CRi rate (*p* = 0.07), whereas mutations in tumor-suppressor genes showed a trend for a lower CR/CRi rate (*p* = 0.07) (Supplementary Table S4). Mutations in signal transduction as well as tumor-suppressor genes showed an inferior OS and EFS from the time of DLI treatment, while all other mutational classes had no prognostic impact in our cohort (Supplementary Table S7). 

Additionally, we evaluated baseline characteristics for their impact on the outcome. We found that adverse cytogenetics (especially, complex karyotype) and extramedullary disease were highly associated with reduced OS and EFS (Supplementary Table S7) in univariate and multivariate analyses (Table 2). Multivariate analysis identified MRC cytogenetic classification, extramedullary manifestation and remission and MRD status to be independently prognostic for EFS and OS (Table 2).

### 3.3. Remission and MRD Status at the Time of First DLI and Its Associations

We first accessed the MRD status at the time of DLIs in patients who received pDLIs (patients in CR/CRi). In CR/CRi, 23 patients were MRD^−^ while 15 patients were MRD^+^ at the time of DLI infusion. Next, we compared clinical baseline characteristics between patients who were in CR/CRi and MRD^−^, in CR/CRi and MRD^+^ and patients not in CR/CRi receiving tDLIs (n = 38) (Table 1). Here, significantly more male compared to female patients were found to be CR/CRi MRD^+^, while other clinical characteristics were comparable between these three groups. Mutations in functional gene classes at diagnosis were also similarly distributed between the groups (Supplementary Table S6).

### 3.4. MRD Conversion Day 30 and Day 90 after DLIs 

We assessed MRD status at day 30 (FU30) and day 90 (FU90) after the first DLI in order to evaluate the MRD conversion rates associated with DLI infusion. Of 23 patients who were CR/CRi MRD^−^ at the time of DLI, none of the patients assessed for MRD at FU30 became MRD^+^ at this point. At FU90, one patient remained in CR/CRi but became MRD^+^ and one patient developed a clinical relapse at this time point (Table 3). Of the 15 patients who were CR/CRi MRD^+^ at the time of DLI, 6 patients (40%) converted to a negative MRD status at FU30 and 11 patients (73%) at FU90, while 7 patients (47%) remained MRD^+^ at FU30 and 2 patients (13%) at FU90. Two patients (13%) of this cohort were not in clinical remission at FU30 and FU90 (Table 3).

Of the 38 patients who were not in CR/CRi at the time of DLI (i.e., receiving tDLIs), 32 (84%) patients remained without CR/CRi at FU30 and 26 patients (68%) at FU90. However, 4 patients (11%) in this cohort were found to be in CR/CRi and MRD^−^ at FU30 and 8 (21%) at FU90. Two patients (5%) were in CR/CRi but MRD^+^ at FU30 and three patients (8%) at FU90. One patient (3%) could not be assessed for MRD at FU90 (Table 3). In summary, a significant number of patients converted from MRD^+^ to MRD^−^ after DLIs with a higher conversion rate at FU90 as compared to FU30, as well as a higher conversion rate in the CR/CRi MRD^+^ patients as compared to patients without CR/CRi at the time of DLI. 

### 3.5. Prognostic Effect of Remission and MRD Status at the Time of First DLI

**Figure 1:**
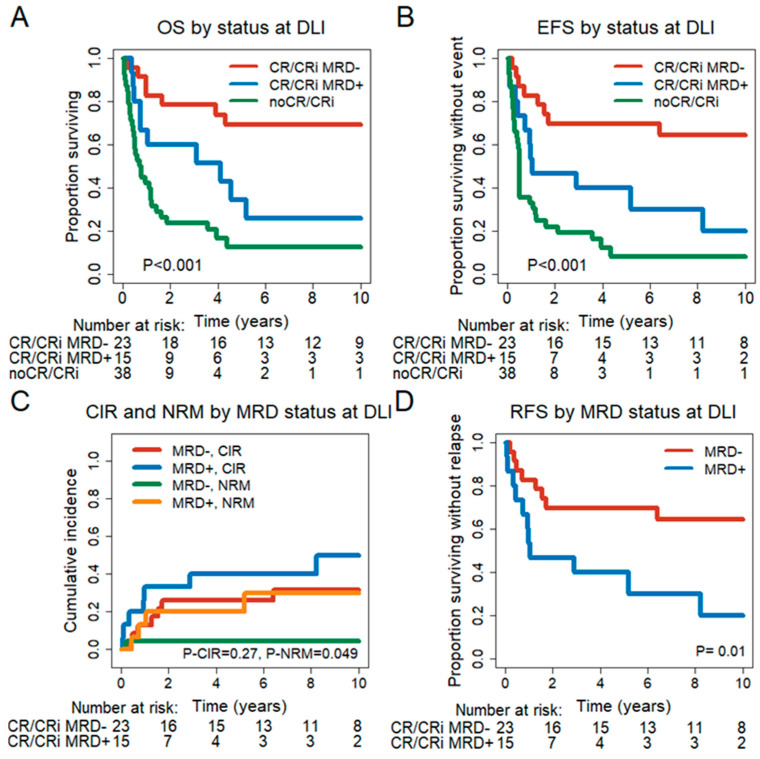
Outcome of patients in CR, CRi or no CR/CRi at the time of DLI. (**A**) OS for patients in CR/CRi and MRD^−^ at DLI (n = 23), in CR/CRi and MRD^+^ (n = 15) and patients not in CR/CRi at DLI (n = 38). (**B**) EFS for patients in CR/CRi at DLI and MRD^−^ (n = 23), in CR/CRi and MRD^+^ (n = 15) and patients not in CR/CRi at DLI (n = 38). (**C**) CIR and NRM for patients in CR/CRi at DLI and MRD^−^ (n = 23) and patients in CR/CRi and MRD^+^ (n = 15). (**D**) RFS for patients in CR/CRi at DLI and MRD^−^ (n = 23) and patients in CR/CRi and MRD^+^ (n = 15).

We next analyzed how remission and MRD status at the time of DLI impacted the outcome after DLI. Patients not in CR/CRi at DLI had a significantly shorter OS (HR = 2.1, 95% CI = 1.01–4.26, *p* = 0.04) and EFS (HR = 1.94, 95% CI = 0.96–3.92, *p* = 0.07) compared to CR/CRi MRD^+^ patients (Figure 1A,B). Patients not in CR/CRi also had a significantly shorter OS (HR = 5.67, 95% CI = 2.45–13.1, *p* < 0.001) and EFS (HR = 5.57, 95% CI = 2.49–12.48, *p* < 0.001) compared to CR/CRi MRD^−^ patients. CR/CRi MRD^+^ patients showed a significantly shorter OS (HR = 3.05, 95% CI = 1.15–8.06, *p* = 0.03) and RFS (HR = 3, 95% CI = 1.2–7.53, *p* = 0.02) compared to CR/CRi MRD^−^ patients (Figure 1A–D). In contrast, CIR (HR = 1.8, 95% CI = 0.65–4.94, *p* = 0.26) and NRM (HR = 6.68, 95% CI = 0.72–62.1, *p* = 0.1) were not significantly different. In summary, remission and MRD status at the time of DLI treatment were highly prognostic for survival outcomes.

### 3.6. Prognostic Effect of MRD Status in CR/CRi Patients 30 and 90 Days after DLI

As MRD status prior to DLI had a significant impact on the outcome after DLI, we were interested in whether MRD status at FU30 and FU90 after the first DLI was prognostic in patients who were in CR/CRi at that time point. 

**Figure 2:**
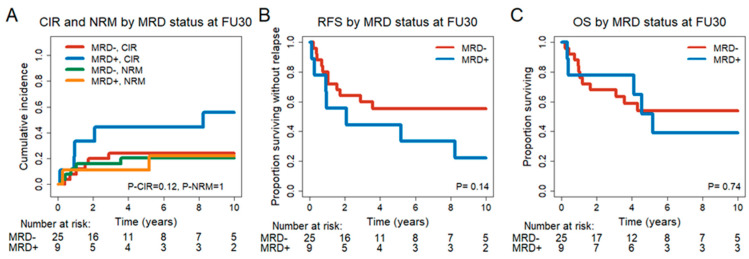
Outcome of patients in CR, CRi or no CR/CRi at 30 days after DLI (FU30). (**A**) CIR and NRM for MRD- (n = 26) and MRD^+^ (n = 9) patients. (**B**) RFS for MRD^−^ (n = 26) and MRD^+^ (n = 9) patients. (**C**) OS for MRD^−^ (n = 26) and MRD^+^ (n = 9) patients.

Of 34 patients in CR/CRi at FU30, 9 patients were MRD^+^, and 25 patients were MRD^−^. A comparison of clinical characteristics showed that MRD^+^ patients had less de novo AML and more sAML, tAML or MDS/AML, a worse HCT-CI score and a lower white blood cell (WBC) count at diagnosis (Supplementary Table S8). Treatment- and transplant-associated characteristics were similarly distributed between the two groups, except for fewer patients with acute GvHD after DLI in the MRD^+^ group (Supplementary Table S9*)*. In addition, mutational classes at diagnosis were similarly distributed (Supplementary Table S10). We observed a trend for an improved CIR (HR = 2.64, 95% CI = 0.86–8.13, *p* = 0.09) in MRD^−^ patients (Figure 2). No prognostic effect of the FU30 MRD status was observed in univariate analysis for NRM (HR = 1.04, 95% CI = 0.22–4.88, *p* = 0.96), RFS (HR = 2.0, 95% CI = 0.77–5.17, *p* = 0.15) and OS (HR = 1.2, 95% CI = 0.42–3.46, *p* = 0.737) (Figure 2, Supplementary Table S11). Baseline characteristics were evaluated as potential biomarkers of outcome. Significant variables from the univariate analysis are given in Supplementary Table S11.

In multivariate analysis, complex karyotype and MRD status at FU30 were predictive for CIR and RFS, and in addition, CMV serostatus for RFS. Extramedullary manifestation at diagnosis and age at DLI were independently predictive factors for NRM, whereas extramedullary manifestation, MRC cytogenetic classification and CMV serostatus were predictive for OS (Table 3).

**Figure 3:**
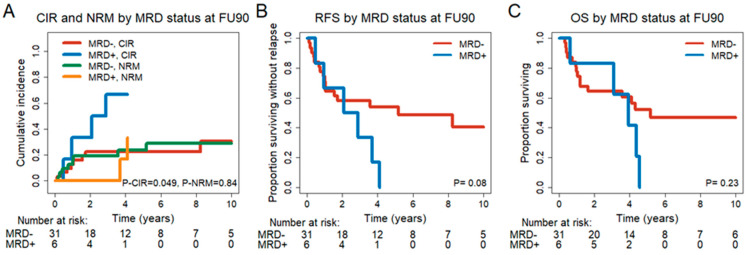
Outcome of patients in CR, CRi or no CR/CRi at 90 days after DLI (FU90) (**A**) CIR and NRM for MRD^−^ (n = 31) and MRD^+^ (n = 6) patients. (**B**) RFS for MRD^−^ (n = 31) and MRD^+^ (n = 6) patients. (**C**) OS for MRD^−^ (n = 31) and MRD^+^ (n = 6) patients.

Next, we investigated the influence of MRD status 90 days after DLI in CR/CRi patients. Of 37 patients in CR/CRi at FU90, 6 patients were FU90 MRD^+^, and 31 patients were MRD^−^. Baseline characteristics (Supplementary Table S12), transplant-associated and genetic characteristics were similarly distributed between the two groups (Supplementary Tables S13 and S14). More MRD^+^ patients had a mutation in a myeloid transcription factor at diagnosis (*p* = 0.017) (Supplementary Table S14). FU90 MRD^+^ status was prognostic for a higher CIR (HR = 3.02, 95% CI = 1.02–8.94, *p* = 0.047) with a trend for RFS (HR = 2.27, 95% CI = 0.87–5.92, *p* = 0.095) but not for NRM (HR = 1.13, 95% CI = 0.3–4.27, *p* = 0.86) and OS (HR = 1.86, 95% CI = 0.67–5.18, *p* = 0.236) in univariate analysis (Figure 3, Supplementary Table S15).

Baseline characteristics were evaluated as potential biomarkers of outcome. Significant variables from the univariate analysis are given in Supplementary Table S15. The multivariate analysis showed MRD status at FU90 and MRC cytogenetic classification to be predictive for CIR (Table 3). MRD status at FU90, complex karyotype and extramedullary manifestation were predictive for RFS (Table 3). MRC cytogenetic classification, CMV serostatus and extramedullary manifestation were predictive for OS. An extramedullary manifestation at diagnosis and age at DLI were predictive for NRM (Table 3). In summary, molecular MRD status 30 and 90 days after DLI treatment is independently prognostic for CIR and RFS, but not for OS. 

### 3.7. Impact of DLIs on MRD^−^ Patients 

Next, we were interested in whether MRD^−^ patients in CR/CRi have benefited from DLI treatment compared to MRD^−^ patients not treated with DLIs. Therefore, we compared in an exploratory analysis CR/CRi MRD^−^ patients at the time of DLI (n = 23) with a control cohort who did not receive DLIs after alloHCT (n = 68). MRD was measured at a similar time point after alloHCT in the control cohort compared to the DLI cohort (median 6 months vs. 5.1 months).

**Figure 4:**
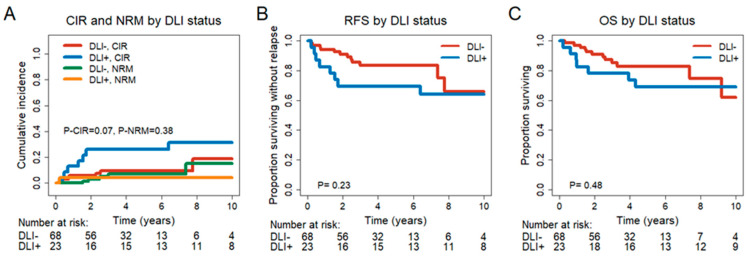
Outcome of patients in CR or CRi who did (n = 23) or did not (n = 68) receive DLIs. (**A**) CIR and NRM by competing risk analysis for patients without DLI (n = 68) and MRD^−^ patients with DLI (n = 23). (**B**) RFS for patients without DLI (n = 68) and MRD^−^ patients with DLI (n = 23). (**C**) OS for patients without DLI (n = 68) and MRD^−^ patients with DLI (n = 23).

Baseline characteristics were similarly distributed between the two groups. However, patients from the control cohort more often had ECOG performance status 1 and 2 at diagnosis and less often had an *FLT3-ITD* mutation compared to DLI-treated patients (Supplementary Table S16). Transplantation-associated characteristics were similar between the patient groups, while patients with DLI more often received grafts from female donors (*p* = 0.02) and experienced less often acute (*p* < 0.001) and chronic GvHD (*p* < 0.001) after alloHCT/before DLI treatment (Supplementary Table S17). No significant differences in CIR (HR = 2.69, 95% CI = 0.9–8.02, *p* = 0.076), NRM (HR = 0.42, 95% CI = 0.037–4.7, *p* = 0.48), RFS (HR = 1.75, 95% CI= 0.7–4.4, *p* = 0.23) and OS (HR = 1.41, 95% CI = 0.54–3.68, *p* = 0.48) could be observed between the DLI and the non-DLI groups (Figure 4).

## 4. Discussion

Little is known about the prognostic role of MRD status and molecular markers in the setting of DLI treatment of AML patients after alloHCT. 

This study reveals that mutations in signal transduction, as well as tumor-suppressor genes, were associated with inferior OS and EFS after DLI treatment. This is in line with published data showing that mutations in tumor-suppressor genes and mutations in signal transduction genes are adverse markers for outcome after chemotherapy as well as alloHCT [19,25,26]. However, in our analysis, these mutations were not independently prognostic for poor outcome after DLI. This might be due to the small sample size but could also indicate that other factors play a more significant prognostic role in the setting of DLI treatment. Here, we identified adverse cytogenetics and extramedullary disease as independent risk factors for poor outcome after DLI. Furthermore, our data also reveal that remission and MRD status at the time of DLI treatment are independently prognostic for survival outcomes after DLI. We were able to demonstrate that MRD^−^ patients prior to DLI showed significantly better OS, EFS and RFS. These results are consistent with published results outlining that MRD status prior to alloHCT is highly prognostic for relapse and survival after alloHCT [15,27,28]. Moreover, studies analyzing the impact of MRD post-transplant observed a significant impact on MRD status. NGS-MRD^+^ 21 days after alloHCT was associated with an increased risk of relapse and worse OS [29], and flow cytometry MRD^+^ patients 30 days after alloHCT had a higher relapse incidence and shorter OS [30]. Zhao et al. described MRD detected by flow cytometry or *WT1* expression within one year after alloHCT as an independent risk factor for post-transplant relapse [31]. Therefore, we were interested to see how DLIs influence MRD status at days 30 and 90 after DLI infusions. Interestingly, we observed a high percentage of patients converting from MRD^+^ prior to DLI to MRD^−^ after DLI. Specifically, upon DLI treatment, 11 (73%) out of 15 MRD^+^ patients converted to MRD^−^ and 12 (32%) out of 38 patients without remission at the time of DLI achieved remission at FU90 after DLI, of which 8 (21%) were MRD^−^, 3 (8%) MRD^+^ and 1 (3%) not assessable for MRD. This underscores the efficacy of DLIs in MRD^+^ patients [32,33,34,35]. But it also shows that an MRD^−^ status is more likely achieved after DLI treatment in those patients with low leukemia burden because we observed fewer patients with active disease turning MRD^−^ (21%) as compared to patients in CR/CRi who were MRD^+^ prior to DLI (73%). This is in line with previous observations describing a prognostic advantage after DLI treatment for low leukemic burden [5,11,36].

In the literature, the 3-year OS for prophylactic and preemptive DLIs varies between 40% and 80% [7,32,33,35,37,38]. Rettig et al. observed a median 2-year OS of 64% for preemptive DLI-treated patients [38]. Schmid et al. described a moderate efficacy of prophylactic DLIs [39]. A benefit in OS (5-year OS 69.8% vs. 40.2%, *p* = 0.027) was only observed for high-risk AML patients but not for standard-risk AML patients compared to a control cohort [39]. However, it has been challenging to monitor molecular response to DLIs. We show that for patients who become MRD^−^ after DLI treatment, this can have prognostic implications. At FU30, MRD status and complex karyotype were independently prognostic for CIR and RFS, and in addition, CMV serostatus for RFS. MRD status at FU90 and MRC cytogenetic classification were independently predictive for CIR. Furthermore, MRD status at FU90 and complex karyotype and extramedullary manifestation were predictive for RFS. Therefore, our data show that molecular MRD status after DLI treatment is independently prognostic for CIR and RFS and thus might become useful for monitoring the response to DLIs. 

The most concerning side effect of DLI treatment is GvHD. In our cohort, 18% of patients developed acute GvHD after the first DLI and 43% after several DLIs. Also, 11% of our patients developed chronic GvHD after the first DLI and 39% after several DLIs, which is similar to previous reports. Two studies described an acute GvHD rate of less than 12% demonstrating a good tolerance of DLIs [37,38,39,40,41]. Other studies observed 17–44% of patients developing acute GvHD [7,13,34,42,43,44,45]. Chronic GvHD is reported in 17–59% of patients [7,42,43,44]. Currently, it is difficult to decide after the first DLI infusion whether to continue treatment with further infusions, which can increase the risk for severe GvHD. It would be ideal to have an early marker for the efficacy of DLI treatment so that we could continue or stop treatment after the first infusions based on the reduction in leukemic burden. Given the lower risk of GvHD after the first DLI infusion and the increasing risk with additional DLI treatments, MRD status after the first DLI infusions might help to select those patients who benefit (i.e., those with a reduction in MRD) or do not benefit from further DLI treatment. Therefore, MRD status after the first DLI infusion might become a helpful tool for the decision to continue DLI infusions or not after further validation. Finally, the therapeutic role of DLIs in MRD^−^ patients is unclear. We compared MRD^−^ patients with similar characteristics at similar time points after alloHCT regarding their outcome with and without DLI treatment. Overall, similar outcomes were observed between patients with or without DLI treatment. However, a prospective trial is necessary to clarify the role of DLIs in MRD-negative patients. 

Our study is limited by the relatively small patient number, and confirmation in a larger cohort is desirable. Since this study was performed retrospectively, not all patients had available samples at both investigated time points after DLI, introducing sampling bias. The treatment period spans a relatively long time from 1998 to 2018 and may add bias to supportive and antileukemic treatment. Some patients who were not in CR/CRi at the time of DLIs received additional treatments besides DLIs, which may confound the effect of DLIs. However, the MRD conversion at 90 days in CR/CRi MRD^+^ patients was achieved in 8 of 11 patients without additional treatments, supporting the therapeutic effect of DLIs in our cohort. 

## 5. Conclusions

In conclusion, we show that a low disease burden at the time of DLI is a favorable prognostic factor for response to DLIs and that MRD negativity 90 days after DLI is associated with a lower CIR and longer RFS in multivariate analysis. Furthermore, our data question the role of prophylactic DLIs in MRD^−^ patients and suggest that MRD status might be helpful for selecting patients who benefit the most from DLI treatment.

## Figures and Tables

**Table 1:** Baseline characteristics of patients who were in CR/CRi and MRD^−^ at DLI, patients who were in CR/CRi and MRD^+^ at DLI and patients who were not in CR/CRi at DLI.

Characteristic	All (n = 76)	CR/CRi MRD^−^ (n = 23)	CR/CRi MRD^+^ (n = 15)	no CR/CRi at DLI (n = 38)	*p*	*p* (MRD^−^ vs. MRD^+^)
Age at diagnosis					0.19	0.73
Median (years)	52.9	50.7	52.8	55.9		
Range (years)	17.4–67.5	18.3–67.5	30–66.9	17.4–66		
Patient sex					0.049	0.013
Male—no. (%)	42 (55)	9 (39)	12 (80)	21 (55)		
Female—no. (%)	34 (45)	14 (61)	3 (20)	17 (45)		
Diagnosis					0.69	0.46
De novo AML—no. (%)	44 (58)	15 (65)	8 (53)	21 (55)		
sAML/tAML/MDS/AML—no. (%)	32 (42)	8 (35)	7 (47)	17 (45)		
Extramedullary manifestation at diagnosis					0.74	0.82
Yes—no. (%)	8 (11)	2 (9)	1 (7)	5 (13)		
No—no. (%)	68 (89)	21 (91)	14 (93)	33 (87)		
FAB subtype					0.51	0.34
M0 + M1 + M2—no. (%)	29 (38)	11 (48)	5 (33.3)	13 (34)		
M4 + M5 + M6 + M7—no. (%)	22 (29)	5 (22)	5 (33.3)	12 (32)		
Not classifiable—no. (%)	25 (33)	7 (30)	5 (33.3)	13 (34)		
FLT3-ITD status at diagnosis					0.4	0.23
Mutated—no. (%)	16 (21)	7 (30)	2 (13)	7 (18)		
Wild type—no. (%)	59 (78)	16 (70)	13 (87)	30 (79)		
Missing—no. (%)	1 (1)	0 (0)	0 (0)	1 (3)		
FLT3-TKD status at diagnosis					0.2	0.07
Mutated—no. (%)	4 (5)	0 (0)	2 (13)	2 (5)		
Wild type—no. (%)	72 (95)	23 (100)	13 (87)	36 (95)		
Complex karyotype					0.96	0.98
Yes—no. (%)	11 (15)	3 (13)	2 (13)	6 (16)		
No—no. (%)	64 (84)	19 (83)	13 (87)	32 (84)		
Missing—no. (%)	1 (1)	1 (4)	0 (0)	0 (0)		
Monosomal karyotype					0.66	0.61
Yes—no. (%)	10 (13)	3 (13)	3 (20)	4 (11)		
No—no. (%)	65 (86)	19 (83)	12 (80)	34 (89)		
Missing—no. (%)	1 (1)	1 (4)	0 (0)	0 (0)		
2022 ELN risk group					0.77	0.85
Favorable + Intermediate—no. (%)	31 (41)	10 (43)	7 (47)	14 (37)		
Adverse—no. (%)	45 (59)	13 (57)	8 (53)	24 (63)		
MRC Grimwade					0.73	0.48
Favorable + Intermediate—no. (%)	56 (74)	17 (74)	10 (67)	29 (76)		
Adverse—no. (%)	19 (25)	5 (22)	5 (33)	9 (24)		
Missing—no. (%)	1 (1)	1 (4)	0 (0)	0 (0)		
ECOG performance status at diagnosis					0.67	0.75
ECOG 0—no. (%)	70 (92)	22 (96)	14 (93)	34 (89)		
ECOG 1—no. (%)	6(8)	1 (4)	1 (7)	4 (11)		
HCT–CI at diagnosis					0.86	0.65
0–2—no. (%)	67 (88)	21 (91)	13 (87)	33 (87)		
>2—no. (%)	9 (12)	2 (99	2 (13)	5 (13)		
WBC count at diagnosis					0.29	0.4
Median—(×10^9^/L)	8	23.9	2.5	8.7		
Range—(×10^9^/L)	0.9–115.2	1.2–115.2	1.4–106.8	0.9–70.6		
Missing—no. (%)	26 (34)	6 (26)	3 (20)	17 (45)		
Hemoglobin at diagnosis					0.78	0.43
Median—g/dL	9.8	9.8	9.6	10.7		
Range—g/dL	5–15	5–14.4	7.9–12.3	5.2–15		
Missing—no. (%)	26 (34)	6 (26)	3 (20)	17 (45)		
Platelet count at diagnosis					0.77	0.93
Median—(×10^9^/L)	61	53.5	50.5	73		
Range—(×10^9^/L)	7–1104	7–212	23–1104	14–469		
Missing—no. (%)	27 (36)	7 (30)	3 (20)	17 (45)		
Blasts in PB at diagnosis					0.15	0.45
Median—%	19.4	67	19.5	11		
Range—%	0–93	0–93	0–82	0–86		
Missing—no. (%)	29 (38)	8 (35)	3 (20)	18 (47)		
Blasts in BM at diagnosis					0.92	0.9
Median—%	48.4	40	46.7	50		
Range—%	5–95	20–95	20–95	5–90		
Missing—no. (%)	44 (58)	15 (65)	6 (40)	23 (61)		

Abbreviations: BM, bone marrow; CMV, cytomegalovirus; CR, complete remission; CRi, CR with incomplete hematological recovery; ECOG, Eastern Cooperative Oncology Group; ELN, European LeukemiaNet; FAB, French–American–British; HCT-CI, Hematopoietic Cell Transplantation-specific Comorbidity Index; ITD, internal tandem duplication; MDS, myelodysplastic syndrome; MRC, Medical Research Council; PB, peripheral blood; sAML, secondary AML; tAML, therapy-related AML; WBC, white blood cell.

**Table 2:** Univariate and multivariate analysis for CIR, NRM, RFS, EFS and OS.

		Univariate Analysis Results			Multivariate Analysis Results		
Endpoint, Cohort	Variables in the Model	HR	95% CI	*p*	HR	95% CI	*p*
EFS, n = 76 Before DLI	Extramedullary manifestation at diagnosis, yes vs. no	2.48	1.11–5.57	0.028	2.41	1.06–5.49	0.036
	MRC Grimwade, adverse vs. favorable + intermediate	2.06	1.12–3.77	0.020	1.88	1.02–3.47	0.044
	No response or CR/CRi MRD^+^ vs. CR/CRi MRD^−^	4.67	2.16–10.7	<0.001	5.05	2.22–11.48	<0.001
OS, n = 76 Before DLI	Extramedullary manifestation at diagnosis, yes vs. no	2.89	1.28–6.56	0.011	3.15	1.35–7.36	0.008
	Complex karyotype, yes vs. no	2.35	1.27–4.36	0.007	2.34	1.25–4.35	0.007
	No response or CR/CRi MRD^+^ vs. CR/CRi MRD^−^	4.67	2.16–10.07	<0.001	5.33	2.23–12.76	<0.001
CIR, n = 34 CR/CRi FU30	Complex karyotype, yes vs. no	3.43	0.87–13.56	0.078	6.72	1.35–33.41	0.02
	FU30 MRD^+^ vs. MRD^−^	2.64	0.86–8.13	0.09	4.6	1.21–17.5	0.025
NRM, n = 34 CR/CRi FU30	Pre DLI median age, ≤54 vs. >54	3.78	0.76–18.91	0.105	7.33	1.05–51.17	0.045
	Extramedullary manifestation at diagnosis, yes vs. no	5.21	0.89–30.41	0.066	13.02	3.32–51.13	<0.001
RFS, n = 34 CR/CRi FU30	Complex karyotype, yes vs. no	3.47	1.08–11.16	0.037	4.45	1.25–15.8	0.021
	CMV status alloHCT1	2.57	0.84–7.87	0.098	3.27	1.0–10.67	0.049
	FU30 MRD^+^ vs. MRD	2	0.77–5.17	0.154	3.91	1.31–11.65	0.015
OS, n = 34 CR/CRi FU30	Extramedullary manifestation at diagnosis, yes vs. no	5.1	1.39–18.67	0.014	22.69	3.77–136.46	0.001
	MRC Grimwade, adverse vs. favorable + intermediate	2.87	1.00–8.26	0.05	4.56	1.36–15.34	0.014
	CMV status alloHCT1	3.19	0.90–11.29	0.072	6.13	1.34–28.06	0.019
CIR, n = 37 CR/CRi FU90	MRC Grimwade, adverse vs. favorable + intermediate	2.68	0.84–8.53	0.095	4.02	1.14–14.22	0.031
	FU90 MRD^+^ vs. MRD^−^	3.02	1.02–8.94	0.047	4.66	1.42–15.31	0.011
NRM, n = 37 CR/CRi FU90	Pre DLI median age, ≤54 vs. >54	5.79	1.24–27.10	0.026	8.4	1.6–44.04	0.012
	Extramedullary manifestation at diagnosis, yes vs. no	4.78	1.10–20.87	0.037	8.71	3.22–23.57	<0.001
RFS, n = 37 CR/CRi FU90	Extramedullary manifestation at diagnosis, yes vs. no	3.59	1.19–10.86	0.024	7.36	1.38–39.1	0.019
	Complex karyotype, yes vs. no	3.17	1.02–9.90	0.047	6.79	1.86–24.81	0.004
	FU90 MRD^+^ vs. MRD	2.27	0.87–5.92	0.095	4.53	1.93–10.66	0.001
OS, n = 37 CR/CRi FU90	Extramedullary manifestation at diagnosis, yes vs. no	3.91	1.27–12.03	0.017	9.67	2.54–36.79	0.001
	MRC Grimwade, adverse vs. favorable + intermediate	2.64	1.02–6.82	0.045	4.27	1.46–12.48	0.008
	CMV status alloHCT1, other vs. D + P neg.	3.01	0.99–9.11	0.052	4.26	1.29–14.07	0.017

Abbreviations: alloHCT, allogeneic hematopoietic cell transplantation; CR, complete remission; CRi, CR with incomplete hematological recovery; D + P, donor and patient; EFS, event-free survival; FU30/90, follow-up time point 30/90 days after DLI; MRD^−^, MRD negative; MRD^+^, MRD positive; NRM, non-relapse mortality; OS, overall survival; RFS, relapse-free survival.

**Table 3:** MRD and remission status before DLI and on day 30 (FU30) and day 90 (FU90) after DLI *.

Remission + MRD Status pre-DLI	n = 76	Remission and MRD Status FU30		Remission and MRD Status FU90	
CR/CRi MRD^−^	23 (30%)	CR/CRi MRD^−^	15 (65%)	CR/CRi MRD^−^	12 (52%)
		CR/CRi MRD^+^	0 (0%)	CR/CRi MRD^+^	1 (4.5%)
		MRD not assessable	8 (35%)	MRD not assessable	9 (39%)
		No CR/CRi	0 (0%)	No CR/CRi	1 (4.5%)
CR/CRi MRD^+^	15 (20%)	CR/CRi MRD^−^	6 (40%)	CR/CRi MRD^−^	11 (73.3%)
		CR/CRi MRD^+^	7 (47%)	CR/CRi MRD^+^	2 (13.3%)
		MRD not assessable	0 (0%)	MRD not assessable	0 (0)
		No CR/CRi	2 (13%)	No CR/CRi	2 (13.3%)
Non-CR/CRi	38 (50%)	CR/CRi MRD^−^	4 (11%)	CR/CRi MRD^−^	8 (21%)
		CR/CRi MRD^+^	2 (5%)	CR/CRi MRD^+^	3 (8%)
		CR/CRi MRD not assessed	0 (0%)	CR/CRi MRD not assessed	1 (3%)
		No CR/CRi	32 (84%)	No CR/CRi	26 (68%)

Abbreviations: CR, complete remission; CRi, CR with incomplete hematological recovery; FU30/90, follow-up time point 30/90 days after DLI. * The reason for non-assessment was a lack of sample availability, but patients were in CR/CRi. Patients not in CR/CRi at DLI include 4 patients with undetectable MRD.

## Data Availability

All data are available from the corresponding author upon request.

## Supplementary Materials

The following supporting information can be downloaded at https://www.mdpi.com/article/10.3390/cancers15153911/s1, Supplementary Figure S1: Mutation signatures were identified using the myeloid panel. MRD was investigated before DLI, 30 days (FU30), and 90 days (FU90) after DLI using error-corrected next-generation sequencing (NGS-MRD). Supplementary Figure S2: Consort diagram showing remission and MRD status at time of DLI, 30 days and 90 days after DLI. DTA-genes were excluded as MRD markers. Patients not in CR/CRi at DLI include 4 patients without molecular marker (NGS^−^). Supplementary Table S2: Genes included in our custom TruSight Myeloid Panel and Illumina custom enrichment panel (Nextera Flex for enrichment) (based on GRCh37/hg19)Supplementary Table S3: Genes included in mutational classes. Supplementary Table S4: Treatment- and transplantation-associated characteristics of patients who were in CR/CRi and MRD^−^ at DLI, patients who were in CR/CRi and MRD^+^ at DLI and patients who were not in CR/CRi at DLI. Supplementary Table S5: Association of mutational classes identified at diagnosis with CR/CRi status 90 days after DLI (FU90). Supplementary Table S6: Frequency of genes used for NGS-MRD analysis and limit of detection (LOD) by gene. Supplementary Table S7: Mutational groups at diagnosis of patients who were in CR/CRi and MRD^−^ at DLI, patients who were in CR/CRi and MRD^+^ at DLI and patients who were not in CR/CRi at DLI. Supplementary Table S8: Univariate analysis of all considered variables for EFS and OS for all patients (n = 76). See excel file “Supplementary Table S7”. Supplementary Table S9: Baseline characteristics of patients in CR/CRi at FU30. Supplementary Table S10: Treatment- and transplantation-associated characteristics of patients in in CR/CRi at FU30. Supplementary Table S11: Molecular characteristics at diagnosis of patients CR/CRi at FU30. Supplementary Table S12: Univariate analysis of all considered variables for CIR, NRM, RFS and OS for patients in CR/CRi at FU30 (n = 34). See excel file “Supplementary Table S11”. Supplementary Table S13: Baseline characteristics at diagnosis of patients CR/CRi at FU90. Supplementary Table S14: Treatment- and transplant-associated characteristics of patients in CR/CRi at FU90. Supplementary Table S15: Molecular characteristics at diagnosis of patients in CR/CRi at FU90. Supplementary Table S16: Univariate analysis of all considered variables for CIR, NRM, RFS and OS for patients in CR/CRi at FU90 (n = 37). See excel file “Supplementary Table S15”. Supplementary Table S17: Baseline characteristics of patients who were MRD^−^ and in CR/CRi before DLI vs. a control cohort without DLI and MRD^−^ after alloHCT. Supplementary Table S18: Treatment- and transplant-associated characteristics of patients who were MRD^−^ and in CR/CRi before DLI and a control cohort without DLI and MRD^−^ after alloHCT.
