# Supplementary material for: MRD as Biomarker for Response to Donor Lymphocyte Infusion after Allogeneic Hematopoietic Cell Transplantation in Patients with AML
Source: Cancers (Basel). 2023 Aug 1;15(15):3911. doi: 10.3390/cancers15153911 (PMC10416875; doi:10.3390/cancers15153911)
Supplement: Supplementary file 1 [file cancers-15-03911-s001.zip › cancers-2492393-supplementary.pdf]

MRD as biomarker for response to donor lymphocyte infusion after allogeneic hematopoietic cell transplantation in patients with AML

Supplementary Data

Supplementary Methods

Supplementary Figure S1: Mutation signatures were identified using the myeloid panel. MRD was investigated before DLI, 30 days (FU30), and 90 days (FU90) after DLI using error-corrected next-generation sequencing (NGS-MRD).

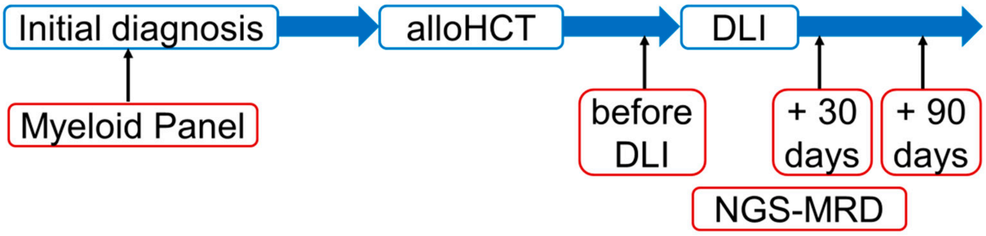

Supplementary Figure S2: Consort diagram showing remission and MRD status at time of DLI, 30 days and 90 days after DLI. DTA-genes were excluded as MRD markers. Patients not in CR/CRi at DLI include 4 patients without molecular marker (NGS^-).

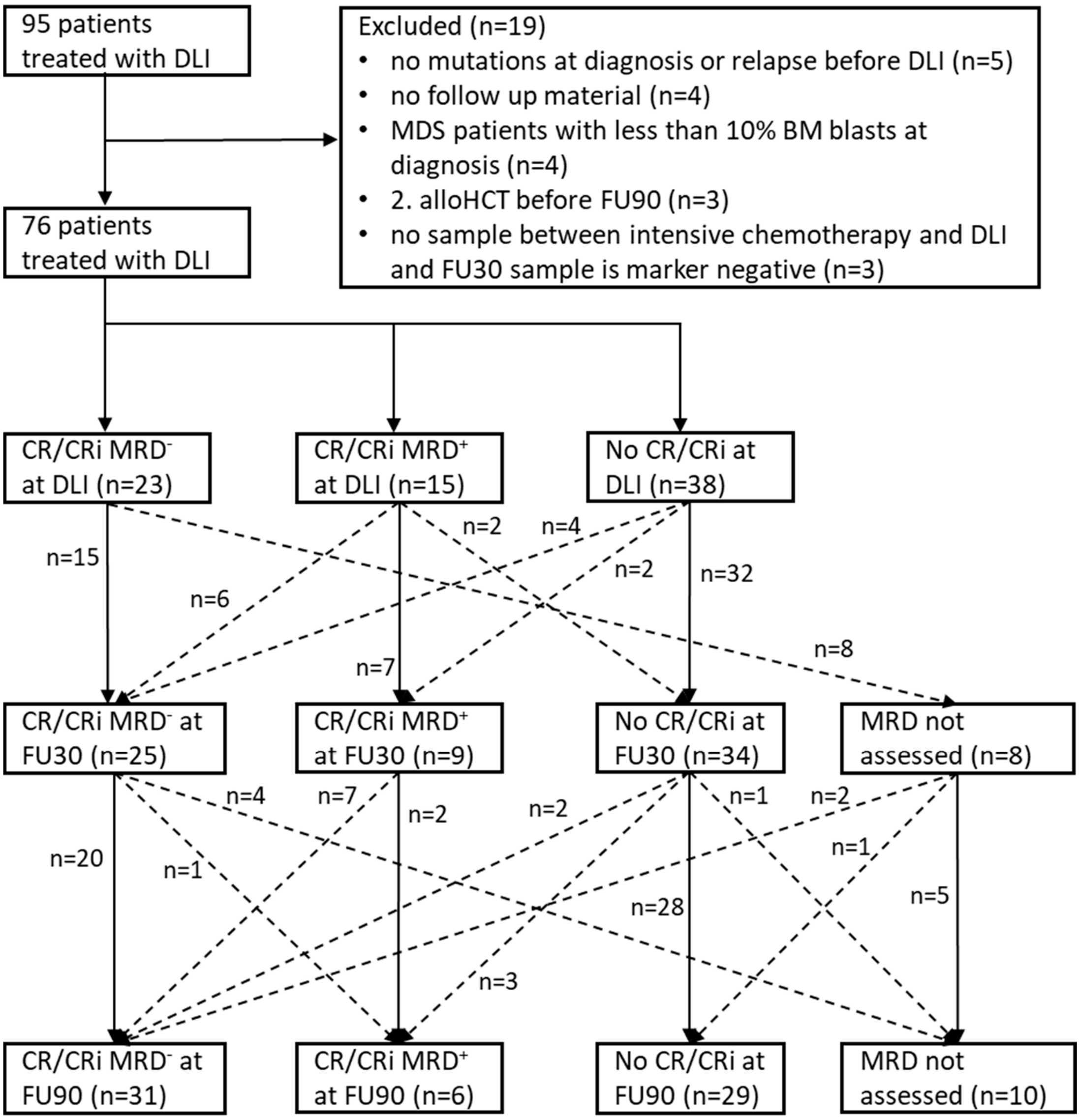

Abbreviations: CR, complete remission; CRi, CR with incomplete hematological recovery; DLI, donor lymphocyte infusion; FU30, follow up 30 days after first DLI; FU90, follow up 90 days after first DLI; MDS, myelodysplastic syndrome; MRD, measurable residual disease.

Supplementary Table S1: Genes included in our custom TruSight Myeloid Panel and Illumina custom enrichment panel (Nextera Flex for enrichment) (based on GRCh37/hg19). *ABCA1* and *GNB1* were exclusive to the Nextera Flex panel, but were not used for this patient co-hort as mutations in these genes were not detected.

**Gene**	**Exons**	**Gene**	**Exons**	**Gene**	**Exons**
ABCA1	2-50	*FLT3*	14-16, 20	*PTPN11*	3, 13
ASXL1	12	*GATA2*	2-6	*RAD21*	complete
ASXL2	11+12	*GNB1*	3-11	*RUNX1*	complete
BCOR	complete	*IDH1*	4	*SETBP1*	4
BCORL1	complete	*IDH2*	4	*SF3B1*	13-16
BRAF	Exon15	*JAK2*	12, 14	*SMC1A*	2, 11, 16, 17
CALR	9	*KDM6A*	complete	*SMC3*	10, 13, 19, 23, 25, 28
CBL	8, 9	*KIT*	2, 8-11, 13, 17	*SRSF2*	1
CEBPA	complete	*KRAS*	2-5	*STAG1*	complete
CSF3R	14-17	*MPL*	10	*STAG2*	complete
CSNK1A1	3, 4	*MYC*	2	*TET2*	3-11
DDX41	complete	*NF1*	complete	*TP53*	2-11
DNMT3A	complete	*NPM1*	12	*U2AF1*	2, 6
ETNK1	3	*NRAS*	2-5	*WT1*	7, 9
ETV6	complete	*PHF6*	complete	*ZBTB7A*	2, 3
EZH2	complete	*PPM1D*	1-6	*ZRSR2*	complete

Supplementary Table S2: Genes included in mutational classes.

**Mutational class**	**Genes**
Epigenetic or chromatin modifier	*ASXL1, ASLX2, DNMT3A, EZH2, IDH1, IDH2, KDM6A, KMT2A, TET2*
Nucleophosmin	*NPM1*
Cohesin complex	*RAD21, SMC1A, SMC3, STAG1, STAG2*
Signal transduction	*CBL, ETNK1, FLT3, JAK2, KIT, KRAS, NF1, NRAS, PTPN11*
Spliceosome	*DDX41, SF3B1, SRSF1, U2AF1, ZBTB7A, ZRSR2*
Myeloid transcription factor	*BCOR, BCORL1, CEBPA, CUX1, ETV6, GATA2, MYC, NOTCH1, PHF6, RUNX1, SETBP1*
Tumor suppressor	*PPM1D, TP53, WT1*

Supplementary Table S3: Treatment- and transplantation-associated characteristics of patients who were in CR/CRi and MRD$^-$ at DLI, patients who were in CR/CRi and MRD$^+$ at DLI and patients who were not in CR/CRi at DLI.

**Characteristic**	**All (n=76)**	**CR/CRi + MRD$^-$ (n=23)**	**CR/CRi + MRD$^+$ (n=15)**	**no CR/CRi at DLI (n=38)**	**p**	**p (MRD$^-$ vs. MRD$^+$)**
Number of chemotherapy cycles before alloHCT1					0.06	0.13
0-1 cycles – no. (%)	11 (14)	3 (13)	5 (33)	3 (8)		
≥2 – no. (%)	65 (86)	20 (87)	10 (67)	35 (92)		
DRI score grouped					0.97	0.96
Low + intermediate – no. (%)	47 (62)	14 (61)	9 (60)	24 (63)		
High + very high – no. (%)	29 (38)	9 (39)	6 (40)	14 (37)		
Remission status at alloHCT1					0.12	0.037
First CR – no. (%)	34 (45)	12 (52)	3 (20)	19 (50)		
CRi – no. (%)	2 (3)	1 (4)	1 (7)	0 (0)		
Partial remission – no. (%)	7 (9)	2 (9)	1 (7)	4 (10)		
Second CR – no. (%)	11 (14)	0 (0)	5 (33)	6 (16)		
No CR/CRi/PR – no. (%)	22 (29)	8 (35)	5 (33)	9 (24)		
Donor match at alloHCT1					0.42	0.95
MRD – no. (%)	25 (33)	10 (43)	6 (40)	9 (24)		
MUD – no. (%)	36 (47)	8 (35)	6 (40)	22 (58)		
MMRD or MMUD – no. (%)	15 (20)	5 (22)	3 (20)	7 (18)		
Conditioning therapy alloHCT1					0.61	0.33
Myeloablative – no. (%)	23 (30)	8 (35)	3 (20)	12 (32)		
Reduced intensity – no. (%)	53 (70)	15 (65)	12 (80)	26 (68)		
Stem cell source at alloHCT1					0.11	0.13
PB – no. (%)	64 (84)	20 (87)	10 (67)	34 (89)		
BM – no. (%)	12 (16)	3 (13)	5 (33)	4 (11)		
Donor sex alloHCT1					0.08	0.026
Male – no. (%)	43 (57)	10 (43)	12 (80)	21 (55)		
Female – no. (%)	33 (43)	13 (57)	3 (20)	17 (45)		
Donor age alloHCT1					0.31	0.045
Median (years)	37	44	33.9	36		
Range (years)	14.9-64.4	14.9-58.7	22.3-58.6	21.1-64.4		
Missing – no. (%)	1 (1)	1 (4)	0 (0)	0 (0)		
Patient age alloHCT1					0.19	0.91
Median (years)	53.2	50.3	53.1	56.9		
Range (years)	17.9-68	18.5-68	30-67	17.9-66		
CMV status alloHCT1					0.83	0.82

Patient neg. /Donor neg. – no. (%)	26 (34)	8 (35)	6 (40)	12 (32)		
Any other constellation – no. (%)	49 (65)	14 (61)	9 (60)	26 (68)		
Missing – no. (%)	1 (1)	1 (4)	0 (0)	0 (0)		
Acute GvHD after alloHCT1 before DLI					0.1	0.06
Yes – no. (%)	23 (30)	3 (13)	6 (40)	14 (37)		
No – no. (%)	53 (70)	20 (87)	9 (60)	24 (63)		
Acute GvHD after alloHCT1 grade					0.72	-
Grade I or II – no. (%)	22 (96)	3 (100)	6 (100)	13 (93)		
Grade III or IV – no. (%)	1 (4)	0 (0)	0 (0)	1 (7)		
Chronic GvHD after alloHCT1 before DLI					0.32	0.14
Yes – no. (%)	14 (18)	2 (9)	4 (17)	8 (21)		
No – no. (%)	62 (82)	21 (91)	11 (73)	30 (79)		
Chronic GvHD after alloHCT1 max. grade					0.67	-
Limited – no. (%)	13 (93)	2 (100)	4 (100)	7 (88)		
Extensive – no. (%)	1 (7)	0 (0)	0 (0)	1 (12)		
Type of first DLI					<0.001	0.06
pDLI – no. (%)	30 (39)	20 (87)	9 (60)	1 (3)		
tDLI – no. (%)	46 (61)	3 (13)	6 (40)	37 (97)		
WBC count at DLI					<0.001	0.99
Median – (x 10^9/l)	3.9	4.9	5.5	1.7		
Range – (x 10^9/l)	0-19	1.4-11.9	1.3-8.2	0-19		
Missing – no. (%)	1 (1)	1 (4)	0 (0)	0 (0)		
Hemoglobin at DLI					<0.001	0.48
Median – g/dL	10.3	11.9	12.5	9.2		
Range – g/dL	6.8-15.3	7.6-15.3	9.2-14.9	6.8-14.2		
Missing – no. (%)	1 (1)	1 (4)	0 (0)	0 (0)		
Platelet count at DLI					<0.001	0.69
Median – (x 10^9/l)	69	127	144	22.5		
Range – (x 10^9/l)	0-274	12-241	31-274	0-192		
Missing – no. (%)	1 (1)	1 (4)	0 (0)	0 (0)		
Blasts PB at DLI					<0.001	1
Median – %	0	0	0	0.9		
Range – %	0-75	0	0	0-75		
Missing – no. (%)	1 (1)	1 (4)	0 (0)	0 (0)		
Blasts BM at DLI					0.034	0.61
Median – %	5	0	3	7		
Range – %	0-80	0-5	0-5	0-80		
Missing – no. (%)	51 (67)	20 (87)	8 (53)	23 (61)		

Time between alloHCT and DLI					0.003	0.036
Median (months)	7.4	5.1	9.1	8.9		
Range – %	3.1-74.7	3.7-21.4	3.1-26	3.3-74.7		
Donor chimerism BM at DLI					0.022	0.21
Median – %	96	99.8	93.5	92		
Range – %	9-100	99-100	78-100	9-100		
Missing – no. (%)	45 (59)	19 (83)	11 (73)	15 (39)		
Donor chimerism PB at DLI					<0.001	0.35
Median – %	99.85	100	99.9	96.8		
Range – %	19-100	77-100	91-100	19-100		
Missing – no. (%)	4 (5)	1 (4)	1 (7)	2 (5)		
Number of DLIs until FU90					0.05	0.08
1 – no. (%)	33 (43)	13 (56)	3 (20)	17 (45)		
2 – no. (%)	25 (33)	8 (35)	9 (60)	8 (21)		
3 – no. (%)	16 (21)	2 (9)	3 (20)	11 (29)		
4 – no. (%)	2 (3)	0 (0)	0 (0)	2 (5)		
DLI starting dose					<0.001	0.91
Median (*10^5 CD3+/kg)	62.5	10	50	100		
Range (*10^5 CD3+/kg)	0.3-210	5-160	0.3-200	0.5-210		
DLI total dose until FU90					0.002	0.34
Median (*10^5 CD3+/kg)	102	55	150	167		
Range (*10^5 CD3+/kg)	0.3-50750	5-600	0.3-1530	16-50750		
Missing – no. (%)	1 (1)	0 (0)	0 (0)	1 (3)		
Age at DLI					0.1	0.84
Median (years)	54.1	52	53.7	57.9		
Range (years)	18.9-68.9	19.1-68.4	30.9-67.7	18.9-68.9		
Donor chimerism PB after DLI					<0.001	0.83
Median – %	99.99	100	100	92.5		
Range – %	12-100	91.2-100	80.9-100	12-100		
Missing – no. (%)	26 (34)	6 (26)	2 (13)	18 (47)		
Acute GvHD after DLI1					0.32	0.13
Yes – no. (%)	14 (18)	6 (26)	1 (7)	7 (18)		
No – no. (%)	62 (82)	17 (74)	14 (93)	31 (82)		
Acute GvHD after DLI1 max grade					0.46	0.21
Grade I or II – no. (%)	8 (57)	4 (67)	0 (0)	4 (57)		
Grade III or IV – no. (%)	6 (43)	2 (33)	0 (0)	3 (43)		
Chronic GvHD after DLI1					0.08	0.05
Yes – no. (%)	8 (11)	5 (22)	0 (0)	3 (8)		
No – no. (%)	68 (89)	18 (78)	15 (100)	35 (92)		
Chronic GvHD after DLI1 max.						-

grade					0.67	
Limited – no. (%)	6 (75)	4 (80)	0 (0)	2 (67)		
Extensive – no. (%)	2 (25)	1 (20)	0 (0)	1 (33)		
AlloHCT2 after DLI1					0.06	0.12
Yes – no. (%)	5 (7)	1 (4)	3 (20)	1 (3)		
No – no. (%)	71 (93)	22 (96)	12 (80)	37 (97)		
CMV reactivation after DLI					0.44	0.34
Yes – no. (%)	13 (17)	4 (17)	1 (7)	8 (21)		
No – no. (%)	62 (82)	19 (83)	14 (93)	29 (76)		
Missing – no. (%)	1 (1)	0 (0)	0 (0)	1 (3)		
Cause of death					<0.001	0.06
Alive – no. (%)	27 (35)	16 (70)	5 (33)	6 (16)		
Relapse – no. (%)	28 (37)	6 (26)	4 (27)	18 (48)		
Progression – no. (%)	2 (3)	0 (0)	0 (0)	2 (5)		
Infection – no. (%)	4 (5)	1 (26)	3 (20)	0 (0)		
Acute GvHD – no. (%)	2 (3)	0 (0)	0 (0)	2 (5)		
Chronic GvHD – no. (%)	4 (5)	0 (0)	2 (13)	2 (5)		
Other/unknown – no. (%)	9 (12)	0 (0)	1 (7)	8 (21)		
Treatment between DLI and FU90					<0.001	0.02
No additional treatment – no. (%)	21 (28)	13 (57)	2 (13)	6 (16)		
Only DLI – no. (%)	28 (37)	10 (42)	10 (67)	8 (21)		
Non-intensive therapy (+DLI) – no. (%)	17 (22)	0 (0)	1 (7)	16 (42)		
Intensive therapy (+DLI) – no. (%)	10 (13)	0 (0)	2 (13)	8 (21)		

Abbreviations: alloHCT, allogeneic hematopoietic cell transplantation; BM, bone marrow; CMV, cytomegalovirus; CR, complete remission; CRi, CR with incomplete haematological recovery; DLI, donor lymphocyte infusion; DRI, disease risk index; FU90, follow up time point 90 days after DLI; GvHD, graft versus host disease; HCT-CI, Hematopoietic Cell Transplantation-specific Comorbidity Index; MRD, matched related donor; MMRD, mismatched related donor; MMUD, mismatched unrelated donor; MUD, matched unrelated donor; PB, peripheral blood; pDLI, preemptive DLI; tDLI, therapeutic DLI; WBC, white blood cell.

Supplementary Table S4: Association of mutational classes identified at diagnosis with CR/CRi status 90 days after DLI (FU90).

**Mutational class at diagnosis**	**All (n=76)**	**noCR/CRi at FU90 (n=29)**	**CR/CRi at FU90 (n=47)**	**p**
Epigenetic modifier				0.41
Mutated – no. (%)	36 (47)	12 (41)	24 (51)	
Wild type – no. (%)	40 (53)	17 (59)	23 (49)	
Nucleophosmin				0.11
Mutated – no. (%)	8 (11)	1 (3)	7 (15)	
Wild type – no. (%)	68 (89)	28 (97)	40 (85)	
Chromatin modifier				0.07
Mutated – no. (%)	5 (7)	0 (0)	5 (11)	
Wild type – no. (%)	71 (93)	29 (100)	42 (89)	
Cohesin complex				0.9
Mutated – no. (%)	11 (14)	4 (14)	7 (15)	
Wild type – no. (%)	65 (86)	25 (86)	40 (85)	
Signal transduction				0.6
Mutated – no. (%)	29 (38)	10 (34)	19 (40)	
Wild type – no. (%)	47 (62)	19 (66)	28 (60)	
Spliceosome				0.31
Mutated – no. (%)	14 (18)	7 (24)	7 (15)	
Wild type – no. (%)	62 (82)	22 (76)	40 (85)	
Myeloid Transcription factor				0.4
Mutated – no. (%)	27 (36)	12 (41)	15 (32)	
Wild type – no. (%)	49 (74)	17 (59)	32 (68)	
Tumor suppressor				0.07
Mutated – no. (%)	20 (26)	11 (38)	9 (19)	
Wild type – no. (%)	56 (72)	18 (62)	38 (81)	
Other				0.43
Mutated – no. (%)	1 (1)	0 (0)	1 (2)	
Wild type – no. (%)	75 (99)	29 (100)	46 (98)	

Supplementary Table S5: Frequency of genes used for NGS-MRD analysis and limit of detection (LOD) by gene.

**Gene**	**Number of MRD-measure-ments (n)**	**Percentage of MRD-measure-ments (%)**	**Number of patients (n)**	**Percentage of patients (%)**	**Median limit of detection (VAF %)**
ASXL1	4	0.86	2	2.6	0.03545
ASXL2	17	3.66	6	7.9	0.0229
BCOR	18	3.87	8	10.5	0.0389
BCORL1	8	1.72	3	3.9	0.0311
CALR	2	0.43	1	1.3	0.0214
CBL	2	0.43	1	1.3	0.0119
CEBPA	5	1.08	2	2.6	0.0332
CSF3R	6	1.29	1	1.3	0.0128
CUX1	3	0.43	1	1.3	1.205
DDX41	6	1.29	1	1.3	0.01965
DNMT3A	43	9.25	18	23.7	0.019
ETNK1	3	0.65	1	1.3	0.0311
ETV6	6	1.29	3	3.9	0.0198
EZH2	11	2.37	4	5.3	0.0224
FLT3	45	9.68	11	14.5	0.01405
GATA2	4	0.86	2	2.6	0.02745
IDH1	9	1.94	3	3.9	0.02015
IDH2	15	3.23	6	7.9	0.0155
JAK2	2	0.43	1	1.3	0.0458
KDM6A	7	1.51	3	3.9	0.0124
KIT	3	0.65	1	1.3	0.0036
KMT2A	3	0.65	1	1.3	0.0341
KRAS	6	1.29	5	6.6	0.021398
NF1	4	0.86	2	2.6	0.0434
NPM1	20	4.30	8	10.5	No LOD*
NRAS	12	2.58	5	6.6	0.0136
PHF6	12	2.58	4	5.3	0.0235
PTPN11	11	2.37	5	6.6	0.0198
RUNX1	29	6.24	11	14.5	0.0233
SF3B1	19	4.09	8	10.5	0.0204
SMC1A	1	0.22	1	1.3	0.03064
SMC3	5	1.08	1	1.3	0.026074088
SRSF2,MFSD11	8	1.72	3	3.9	0.02545
STAG2	17	3.66	7	9.2	0.01365
TET2	35	7.53	11	14.5	0.0206
TP53	31	6.67	12	15.8	0.02605

U2AF1	8	1.72	3	3.9	0.0073
WT1	24	5.16	10	13.2	0.0256
ZBTB7A	1	0.22	1	1.3	0.0238
ZRSR2	1	0.22	1	1.3	0.036
**Total**	**466**	**100**	**178**	**Median 2 genes per patient**	**Median LOD: 0.0221**

*Limit of detection is defined as 10 supporting reads.

Supplementary Table S6: Mutational groups at diagnosis of patients who were in CR/CRi and MRD$^-$ at DLI, patients who were in CR/CRi and MRD$^+$ at DLI and patients who were not in CR/CRi at DLI.

**Mutational class at diagnosis**	**All (n=76)**	**CR/CRi MRD$^-$ (n=23)**	**CR/CRi MRD$^+$ (n=15)**	**no CR/CRi at DLI (n=38)**	**p (all)**	**p (MRD$^-$ vs. MRD$^+$)**
Epigenetic modifier					0.85	0.74
Mutated – no. (%)	36 (47)	11 (48)	8 (53)	17 (45)		
Wild type – no. (%)	40 (53)	12 (52)	7 (47)	21 (55)		
Nucleophosmin					0.33	0.15
Mutated – no. (%)	8 (11)	3 (13)	0 (0)	5 (13)		
Wild type – no. (%)	68 (89)	20 (87)	15 (100)	33 (87)		
Chromatin modifier					0.28	0.53
Mutated – no. (%)	5 (7)	3 (13)	1 (7)	1 (3)		
Wild type – no. (%)	71 (93)	20 (87)	14 (93)	37 (97)		
Cohesin complex					0.57	0.65
Mutated – no. (%)	11 (14)	2 (9)	2 (13)	7 (18)		
Wild type – no. (%)	65 (86)	21 (91)	12 (87)	31 (82)		
Signal transduction					0.4	0.26
Mutated – no. (%)	29 (38)	8 (35)	8 (53)	13 (34)		
Wild type – no. (%)	47 (62)	15 (65)	7 (47)	25 (66)		
Spliceosome					0.35	0.34
Mutated – no. (%)	14 (18)	4 (17)	1 (7)	9 (23)		
Wild type – no. (%)	62 (82)	19 (83)	14 (93)	29 (76)		
Myeloid Transcription factor					0.37	0.22
Mutated – no. (%)	27 (36)	9 (39)	3 (20)	15 (39)		

Wild type – no. (%)	49 (65)	14 (61)	12 (80)	23 (61)		
Tumor suppressor					0.25	0.54
Mutated – no. (%)	20 (26)	5 (22)	2 (13)	13 (34)		
Wild type – no. (%)	56 (74)	18 (78)	12 (87)	25 (66)		

Supplementary Table S7: Univariate analysis of all considered variables for EFS and OS for all patients (n=76). See excel file “Supplementary Table S7”.

Supplementary Table S8: Baseline characteristics of patients in CR/CRi at FU30.

**Characteristic**	**All (n=34)**	**MRD$^-$ FU30 (n=25)**	**MRD$^+$ FU30 (n=9)**	**p**
Age at diagnosis				0.56
Median (years)	51.3	50.1	56.9	
Range (years)	21-66.9	21-66.9	38.9-64.8	
Patient sex				0.86
Male – no. (%)	18 (53)	13 (52)	5 (56)	
Female – no. (%)	16 (47)	12 (48)	4 (44)	
Diagnosis				0.041
De novo AML – no. (%)	21 (62)	18 (72)	3 (33)	
sAML / tAML / MDS/AML – no. (%)	13 (38)	7 (28)	6 (67)	
Extramedullary manifestation at diag-nosis				0.26
Yes – no. (%)	4 (12)	2 (8)	2 (22)	
No – no. (%)	30 (88)	23 (92)	7 (78)	
FAB–subtype				0.26
M0 + M1 + M2 – no. (%)	18 (53)	15 (60)	3 (33)	
M4 + M5 + M6 + M7 – no. (%)	5 (15)	3 (12)	2 (22)	
Not classifiable – no. (%)	11 (32)	7 (28)	5 (45)	
FLT3-ITD status at diagnosis				0.41
Yes – no. (%)	7 (21)	6 (24)	1 (11)	
No – no. (%)	27 (79)	19 (76)	8 (89)	
FLT3-TKD status at diagnosis				0.79
Yes – no. (%)	3 (9)	2 (8)	1 (11)	
No – no. (%)	31 (91)	23 (92)	8 (89)	
Complex karyotype				0.15
Yes – no. (%)	5 (15)	5 (20)	0 (0)	

No – no. (%)	29 (85)	20 (80)	9 (100)	
Monosomal karyotype				0.15
Yes – no. (%)	5 (15)	5 (20)	0 (0)	
No – no. (%)	29 (85)	20 (80)	9 (100)	
2022 ELN risk group				0.5
Favorable + Intermediate – no. (%)	12 (35)	8 (32)	4 (44)	
Adverse – no. (%)	22 (65)	17 (68)	5 (56)	
MRC Grimwade				0.22
Favorable + Intermediate – no. (%)	25 (74)	17 (68)	8 (89)	
Adverse – no. (%)	9 (26)	8 (32)	1 (11)	
ECOG performance status at diagnosis				0.44
ECOG 0 – no. (%)	32 (94)	24 (96)	8 (89)	
ECOG 1 – no. (%)	2 (6)	1 (4)	1 (11)	
HCT–CI at diagnosis				0.019
0-2 – no. (%)	30 (88)	24 (96)	6 (67)	
>2 – no. (%)	4 (12)	1 (4)	3 (33)	
WBC count at diagnosis				0.014
Median – (x 10^9/l)	7.8	23.9	1.9	
Range – (x 10^9/l)	1.2-115.2	1.2-115.2	1.4-2.9	
Missing – no. (%)	9 (26)	6 (24)	3 (33)	
Hemoglobin at diagnosis				0.57
Median – g/dL	9.7	9.8	9.3	
Range – g/dL	6.7-14.4	6.7-14.4	8-10.8	
Missing – no. (%)	9 (26)	6 (24)	3 (33)	
Platelet count at diagnosis				0.45
Median – (x 10^9/l)	46	55	32	
Range – (x 10^9/l)	7-1104	7-1104	20-201	
Missing – no. (%)	9 (26)	6 (24)	3 (33)	
Blasts PB at diagnosis				0.34
Median – %	39.8	67.5	1.5	
Range – %	0-93	0-93	0-70	
Missing – no. (%)	10 (29)	7 (28)	3 (33)	
Blasts BM at diagnosis				0.3
Median – %	48.4	80	40	
Range – %	20-95	20-95	25-47	
Missing – no. (%)	20 (59)	14 (56)	6 (67)	

Abbreviations: BM, bone marrow; CMV, cytomegalovirus; CR, complete remission; CRi, CR with incomplete haematological recovery; ECOG, Eastern Cooperative Oncology Group; ELN, European leukemia net; FAB, French-American-British; HCT-CI, Hematopoietic Cell Transplantation-specific Comorbidity Index; ITD, internal tandem duplication; MDS, Myelodysplas-

tic syndrome; MRC, Medical research council; PB, peripheral blood; sAML, secondary AML; tAML, therapy-related AML; WBC, white blood cell.

Supplementary Table S9: Treatment- and transplantation-associated characteristics of patients in in CR/CRi at FU30.

Characteristic	All (n=34)	MRD$^-$ FU30 (n=25)	MRD$^+$ FU30 (n=9)	p
Number of chemotherapy cycles before alloHCT1				0.21
0-1 cycles – no. (%)	9 (26)	4 (16)	5 (56)	
≥2 – no. (%)	25 (74)	21 (84)	4 (44)	
DRI score grouped				0.31
Low + intermediate – no. (%)	20 (59)	16 (64)	4 (44)	
High + very high – no. (%)	14 (41)	9 (36)	5 (56)	
Remission status at alloHCT1				0.3
First CR – no. (%)	12 (35)	11 (44)	1 (11)	
CRi – no. (%)	2 (6)	2 (8)	0 (0)	
Partial remission – no. (%)	2 (6)	1 (4)	1 (11)	
Second CR – no. (%)	6 (18)	4 (16)	2 (22)	
No CR/Cri/PR – no. (%)	12 (35)	7 (28)	5 (56)	
Donor match at alloHCT1				0.85
MRD – no. (%)	13 (38)	9 (36)	4 (45)	
MUD – no. (%)	14 (41)	11 (44)	3 (33)	
MMRD or MMUD – no. (%)	7 (21)	5 (20)	2 (22)	
Conditioning therapy alloHCT1				0.22
Myeloablative – no. (%)	9 (26)	8 (32)	1 (11)	
Reduced intensity – no. (%)	25 (74)	17 (68)	8 (89)	
Stem cell source at alloHCT1				0.039
PB – no. (%)	27 (79)	22 (88)	5 (56)	
BM – no. (%)	7 (21)	3 (12)	4 (44)	
Donor sex alloHCT1				0.25
Male – no. (%)	21 (62)	14 (56)	7 (78)	
Female – no. (%)	13 (38)	11 (44)	2 (22)	
Donor age alloHCT1				0.98
Median (years)	41.8	42.4	37	
Range (years)	21.1-64.4	22.9-58.7	21.1-64.4	
Missing – no. (%)	1 (3)	1 (4)	0 (0)	
Patient age alloHCT1				0.64

Median (years)	51.7	51	58	
Range (years)	21.5-67	21.5-67	39-65	
CMV status alloHCT1				0.5
Patient neg. /Donor neg. – no. (%)	12 (35)	8 (32)	4 (44)	
Any other constellation – no. (%)	22 (65)	17 (68)	5 (56)	
Acute GvHD after alloHCT1 before DLI				0.59
Yes – no. (%)	9 (26)	6 (24)	3 (33)	
No – no. (%)	25 (74)	19 (76)	6 (67)	
Acute GvHD after alloHCT1 grade				-
Grade I or II – no. (%)	9 (100)	6 (100)	3 (100)	
Grade III or IV – no. (%)	0 (0)	0 (0)	0 (0)	
Chronic GvHD after alloHCT1 before DLI				0.27
Yes – no. (%)	7 (21)	4 (16)	3 (33)	
No – no. (%)	27 (79)	21 (84)	6 (67)	
Chronic GvHD after alloHCT1 max. grade				-
Limited – no. (%)	7 (100)	4 (100)	3 (100)	
Extensive – no. (%)	0 (0)	0 (0)	0 (0)	
Type first DLI				0.89
pDLI – no. (%)	22 (65)	16 (64)	6 (67)	
tDLI – no. (%)	12 (35)	9 (36)	3 (33)	
Remission status before DLI1				0.68
CR/CRi – no. (%)	28 (82)	21 (84)	7 (78)	
No CR/CRi – no. (%)	6 (18)	4 (16)	2 (22)	
WBC count at DLI				0.47
Median – (x 10^9/l)	5	5.2	4.5	
Range – (x 10^9/l)	0.3-11.9	0.3-11.9	0.8-8.2	
Hemoglobin at DLI				0.4
Median – g/dL	11.2	10.7	12.6	
Range – g/dL	7.1-15.3	7.1-15.3	9-14.9	
Platelet count at DLI				0.99
Median – (x 10^9/l)	125.5	121	143	
Range – (x 10^9/l)	12-274	13-231	12-274	
Blasts PB at DLI				1
Median – %	0	0	0	
Range – %	0-4.3	0-4.3	0-1	
Blasts BM at DLI				0.82
Median – %	4	4	25	
Range – %	0-50	0-7	0-50	
Missing – no. (%)	24 (71)	17 (63)	7 (78)	
Time between alloHCT and DLI				0.53
Median (month)	7.7	7.7	9.1	
Range – %	3.1-74.7	3.1-74.7	4.3-22.8	
Donor chimerism BM at DLI				0.18

Median – %	98.3	100	90	
Range – %	37-100	37-100	47-97	
Missing – no. (%)	26 (76)	20 (80)	6 (67)	
Donor chimerism PB at DLI				0.86
Median – %	100	100	100	
Range – %	91-100	95-100	91-100	
Missing – no. (%)	1 (3)	1 (4)	0 (0)	
Number of DLIs until FU90				0.25
1 – no. (%)	15 (44)	13 (52)	2 (22)	
2 – no. (%)	15 (44)	9 (36)	6 (67)	
3 – no. (%)	4 (12)	3 (12)	1 (11)	
DLI starting dose				0.95
Median (*10^5 CD3+/kg)	10	50	10	
Range (*10^5 CD3+/kg)	0.3-210	5-210	0.3-100	
DLI total dose until FU90				0.98
Median (*10^5 CD3+/kg)	60	75	60	
Range (*10^5 CD3+/kg)	0.3-1530	5-1530	0.3-1380	
Age at DLI				0.7
Median (years)	52.3	51.7	58.8	
Range (years)	22.9-68.9	22.9-68.9	40-66.3	
Donor chimerism PB after DLI				0.79
Median – %	100	100	100	
Range – %	86-100	97-100	86-100	
Missing – no. (%)	7 (21)	5 (20)	2 (22)	
Acute GvHD after DLI1				0.05
Yes – no. (%)	8 (24)	8 (32)	0 (0)	
No – no. (%)	26 (76)	17 (68)	9 (100)	
Acute GvHD after DLI1 max grade				-
Grade I or II – no. (%)	5 (63)	5 (63)	0 (0)	
Grade III or IV – no. (%)	3 (37)	3 (37)	0 (0)	
Chronic GvHD after DLI1				0.15
Yes – no. (%)	5 (15)	5 (20)	0 (0)	
No – no. (%)	29 (85)	20 (80)	9 (100)	
Chronic GvHD after DLI1 max. grade				-
Limited – no. (%)	4 (80)	4 (80)	0 (0)	
Extensive – no. (%)	1 (10)	1 (10)	0 (0)	
AlloHCT2 after DLI1				0.1
Yes – no. (%)	3 (9)	1 (4)	2 (22)	
No – no. (%)	31 (91)	24 (96)	7 (78)	
CMV reactivation after DLI				0.72
Yes – no. (%)	5 (15)	4 (16)	1 (11)	
No – no. (%)	29 (85)	21 (84)	8 (89)	
Cause of death				0.05
Alive – no. (%)	18 (53)	14 (56)	4 (45)	

Relapse – no. (%)	8 (23)	6 (24)	2 (22)	
Infection – no. (%)	3 (9)	1 (4)	2 (22)	
Chronic GvHD – no. (%)	2 (6)	2 (8)	0 (0)	
Other/unknown – no. (%)	3 (9)	2 (8)	1 (11)	
Treatment between DLI and FU90				0.12
No additional treatment – no. (%)	13 (38)	12 (48)	1 (11)	
Only DLI – no. (%)	17 (50)	11 (44)	6 (67)	
Non-intensive therapy (+DLI) – no. (%)	1 (3)	1 (4)	0 (0)	
Intensive therapy (+DLI) – no. (%)	3 (9)	1 (4)	2 (22)	
Treatment after FU90				0.16
No – no. (%)	21 (62)	18 (72)	3 (33)	
DLIs only – no. (%)	3 (9)	2 (8)	1 (11)	
Non-intensive treatment – no. (%)	3 (9)	2 (8)	1 (11)	
Intensive treatment – no. (%)	7 (20)	3 (12)	4 (45)	

Abbreviations: alloHCT, allogeneic hematopoietic cell transplantation; BM, bone marrow; CMV, cytomegalovirus; CR, complete remission; CRi, CR with incomplete haematological recovery; DLI, donor lymphocyte infusion; DRI, disease risk index; FU90, follow up time point 90 days after DLI; GvHD, graft versus host disease; HCT-CI, Hematopoietic Cell Transplantation-specific Comorbidity Index; MRD, matched related donor; MMRD, mismatched related donor; MMUD, mismatched unrelated donor; MUD, matched unrelated donor; PB, peripheral blood; pDLI, preemptive DLI; tDLI, therapeutic DLI; WBC, white blood cell.

Supplementary Table S10: Molecular characteristics at diagnosis of patients CR/CRi at FU30.

**Mutational class at diagnosis**	**All (n=34)**	**MRD$^-$ FU30 (n=25)**	**MRD$^+$ FU30 (n=9)**	**p**
Epigenetic modifier				0.18
Mutated – no. (%)	20 (59)	13 (52)	7 (78)	
Wild type – no. (%)	14 (41)	12 (48)	2 (22)	
Nucleophosmin				0.54
Mutated – no. (%)	1 (3)	1 (4)	0 (0)	
Wild type – no. (%)	33 (97)	24 (96)	9 (100)	

Chromatin modifier				0.46
Mutated – no. (%)	5 (15)	3 (12)	2 (22)	
Wild type – no. (%)	29 (85)	22 (88)	7 (78)	
Cohesin complex				0.15
Mutated – no. (%)	6 (18)	3 (12)	3 (33)	
Wild type – no. (%)	28 (82)	22 (88)	6 (67)	
Signal transduction				0.94
Mutated – no. (%)	11 (32)	8 (32)	3 (33)	
Wild type – no. (%)	23 (68)	17 (68)	6 (67)	
Spliceosome				0.55
Mutated – no. (%)	6 (18)	5 (20)	1 (11)	
Wild type – no. (%)	28 (82)	20 (80)	8 (89)	
Myeloid Transcription factor				0.22
Mutated – no. (%)	9 (26)	8 (32)	1 (11)	
Wild type – no. (%)	25 (74)	17 (68)	8 (89)	
Tumor suppressor				0.72
Mutated – no. (%)	5 (15)	4 (16)	1 (11)	
Wild type – no. (%)	29 (85)	21 (84)	8 (89)	

Supplementary Table S11: Univariate analysis of all considered variables for CIR, NRM, RFS and OS for patients in CR/CRi at FU30 (n=34). See excel file "Supplementary Table S11".

Supplementary Table S12: Baseline characteristics at diagnosis of patients CR/CRi at FU90.

**Characteristic**	**All (n=37)**	**MRD$^-$ FU90 (n=31)**	**MRD$^+$ FU90 (n=6)**	**p**
Age at diagnosis				0.71
Median (years)	51.7	51	54.7	
Range (years)	18.3-67.5	18.3-66.9	38.9-67.5	
Patient sex				0.94
Male – no. (%)	18 (49)	15 (48)	3 (50)	
Female – no. (%)	19 (51)	16 (52)	3 (50)	
Diagnosis				0.11
De novo AML – no. (%)	23 (62)	21 (68)	2 (33)	
sAML / tAML / MDS/AML – no. (%)	14 (38)	10 (32)	4 (67)	

Extramedullary manifestation at diagnosis				0.29
Yes – no. (%)	5 (14)	5 (16)	0 (0)	
No – no. (%)	32 (86)	26 (84)	6 (100)	
FAB–subtype				0.22
M0 + M1 + M2 – no. (%)	16 (43)	13 (23)	3 (50)	
M4 + M5 + M6 + M7 – no. (%)	7 (19)	7 (42)	0 (0)	
Not classifiable – no. (%)	14 (38)	11 (35)	3 (50)	
FLT3-ITD status at diagnosis				0.45
Yes – no. (%)	8 (22)	6 (19)	2 (33)	
No – no. (%)	29 (78)	25 (81)	4 (67)	
FLT3-TKD status at diagnosis				0.57
Yes – no. (%)	4 (11)	4 (13)	0 (0)	
No – no. (%)	33 (89)	27 (87)	6 (100)	
Complex karyotype				0.29
Yes – no. (%)	5 (14)	5 (16)	0 (0)	
No – no. (%)	32 (86)	26 (84)	6 (100)	
Monosomal karyotype				0.97
Yes – no. (%)	6 (16)	5 (16)	1 (17)	
No – no. (%)	31 (84)	26 (84)	5 (33)	
2022 ELN risk group				0.83
Favorable + Intermediate – no. (%)	17 (46)	14 (45)	3 (50)	
Adverse – no. (%)	29 (54)	17 (55)	3 (50)	
MRC Grimwade				0.53
Favorable + Intermediate – no. (%)	27 (73)	22 (71)	5 (83)	
Adverse – no. (%)	10 (27)	9 (29)	1 (17)	
ECOG performance status at diagnosis				0.52
ECOG 0 – no. (%)	35 (95)	29 (94)	6 (100)	
ECOG 1 – no. (%)	2 (5)	2 (6)	0 (0)	
HCT–CI at diagnosis				0.81
0-2 – no. (%)	32 (86)	27 (87)	5 (83)	
>2 – no. (%)	5 (14)	4 (13)	1 (17)	
WBC count at diagnosis				0.18
Median – (x 10^9/l)	3.6	7.9	2.2	
Range – (x 10^9/l)	1.4-115.2	1.4-115.2	1.7-5.5	
Missing – no. (%)	10 (27)	9 (29)	1 (17)	
Hemoglobin at diagnosis				0.3
Median – g/dL	9.7	9.5	10.8	
Range – g/dL	5-14.4	5-14.4	8-11.8	
Missing – no. (%)	10 (27)	9 (29)	1 (17)	
Platelet count at diagnosis				0.21
Median – (x 10^9/l)	60	61.5	37	
Range – (x 10^9/l)	7-1104	7-1104	20-469	
Missing – no. (%)	10 (27)	9 (28)	1 (17)	

Blasts PB at diagnosis				0.45
Median – %	18.2	19.6	0	
Range – %	0-93	0-93	0-60	
Missing – no. (%)	11 (30)	10 (32)	1 (17)	
Blasts BM at diagnosis				0.72
Median – %	37.5	43.4	25	
Range – %	20-95	20-95	20-90	
Missing – no. (%)	19 (51)	17 (55)	2 (33)	

Abbreviations: BM, bone marrow; CMV, cytomegalovirus; CR, complete remission; CRi, CR with incomplete haematological recovery; ECOG, Eastern Cooperative Oncology Group; ELN, European leukemia net; FAB, French-American-British; HCT-CI, Hematopoietic Cell Transplantation-specific Comorbidity Index; ITD, internal tandem duplication; MDS, Myelodysplastic syndrome; MRC, Medical research council; PB, peripheral blood; sAML, secondary AML; tAML, therapy-related AML; WBC, white blood cell.

Supplementary Table S13: Treatment- and transplant-associated characteristics of patients in CR/CRi at FU90.

**Characteristic**	**All (n=37)**	**MRD$^-$ FU90 (n=31)**	**MRD$^+$ FU90 (n=6)**	**p**
Number of chemotherapy cycles before alloHCT1				0.57
0-1 cycles – no. (%)	9 (24)	7 (23)	2 (33)	
≥2 – no. (%)	28 (76)	24 (77)	4 (67)	
DRI score grouped				0.8
Low + intermediate – no. (%)	23 (62)	19 (61)	4 (67)	
High + very high – no. (%)	14 (38)	12 (39)	2 (33)	
Remission status at alloHCT1				0.92
First CR – no. (%)	15 (41)	13 (42)	2 (33)	
CRi – no. (%)	1 (3)	1 (3)	0 (0)	
Partial remission – no. (%)	3 (8)	2 (7)	1 (17)	
Second CR – no. (%)	6 (16)	5 (16)	1 (17)	
No CR/Cri/PR – no. (%)	12 (32)	10 (32)	2 (33)	
Donor match at alloHCT1				0.54

MRD – no. (%)	13 (35)	12 (39)	1 (17)	
MUD – no. (%)	16 (43)	13 (42)	3 (50)	
MMRD or MMUD – no. (%)	8 (22)	6 (19)	2 (33)	
Conditioning therapy alloHCT1				0.1
Myeloablative – no. (%)	10 (27)	10 (32)	0 (0)	
Reduced intensity – no. (%)	27 (73)	21 (68)	6 (100)	
Stem cell source at alloHCT1				0.45
PB – no. (%)	29 (78)	25 (81)	4 (67)	
BM – no. (%)	8 (22)	6 (19)	2 (33)	
Donor sex alloHCT1				0.59
Male – no. (%)	21 (57)	17 (55)	4 (67)	
Female – no. (%)	16 (43)	14 (45)	2 (33)	
Donor age alloHCT1				0.015
Median (years)	38.6	42.5	26.7	
Range (years)	20.3-64.4	20.3-64.4	21.1-35.1	
Missing – no. (%)	1 (3)	1 (3)	0 (0)	
Patient age alloHCT1				0.71
Median (years)	52.1	51.3	55.6	
Range (years)	18.5-68	18.5-67	39-68	
CMV status alloHCT1				0.92
Patient neg. /Donor neg. – no. (%)	13 (35)	11 (35)	2 (33)	
Any other constellation – no. (%)	24 (65)	20 (65)	4 (67)	
Acute GvHD after alloHCT1 before DLI				0.7
Yes – no. (%)	10 (27)	8 (26)	2 (33)	
No – no. (%)	27 (73)	23 (74)	4 (67)	
Acute GvHD after alloHCT1 grade				-
Grade I or II – no. (%)	10 (100)	8 (100)	2 (100)	
Grade III or IV – no. (%)	0 (0)	0 (0)	0 (0)	
Chronic GvHD after alloHCT1 before DLI				0.75
Yes – no. (%)	8 (22)	7 (23)	1 (17)	
No – no. (%)	29 (78)	24 (77)	5 (83)	
Chronic GvHD after alloHCT1 max. grade				-
Limited – no. (%)	8 (100)	7 (100)	1 (100)	
Extensive – no. (%)	0 (0)	0 (0)	0 (0)	
Type first DLI				0.83
pDLI – no. (%)	20 (54)	17 (55)	3 (50)	
tDLI – no. (%)	17 (46)	14 (45)	3 (50)	
Remission status before DLI1				0.24
CR/CRi – no. (%)	26 (70)	23 (74)	3 (50)	
No CR/CRi – no. (%)	11 (30)	8 (26)	3 (50)	
WBC count at DLI				0.57
Median – (x 10^9/l)	4.5	4.7	4.1	
Range – (x 10^9/l)	0.3-11.9	0.3-11.9	0.8-7.6	

Hemoglobin at DLI				0.89
Median – g/dL	10.7	10.6	11.9	
Range – g/dL	7.1-15.3	7.1-15.3	8-13.4	
Platelet count at DLI				0.14
Median – (x 10^9/l)	112	143	66.5	
Range – (x 10^9/l)	0-274	0-274	17-141	
Blasts PB at DLI				1
Median – %	0	0	0	
Range – %	0-6	0-5	0-1	
Blasts BM at DLI				0.96
Median – %	4	5	3	
Range – %	0-50	0-50	0-5	
Missing – no. (%)	25 (68)	22 (71)	3 (50)	
Time between alloHCT and DLI				0.93
Median (month)	8.1	7.7	9.5	
Range – %	3.1-74.7	3.7-74.7	3.1-18.9	
Donor chimerism BM at DLI				0.65
Median – %	97	98.3	92	
Range – %	37-100	37-100	90-99	
Missing – no. (%)	26 (70)	23 (74)	3 (50)	
Donor chimerism PB at DLI				0.06
Median – %	99.9	100	27.5	
Range – %	87-100	95.8-100	0.3-100	
Missing – no. (%)	1 (3)	1 (3)	0 (0)	
Number of DLIs until FU90				0.2
1 – no. (%)	13 (35)	10 (32)	3 (50)	
2 – no. (%)	18 (49)	17 (55)	1 (17)	
3 – no. (%)	6 (16)	4 (13)	2 (33)	
DLI starting dose				0.93
Median (*10^5 CD3+/kg)	50	50	27.5	
Range (*10^5 CD3+/kg)	0.3-210	5-210	0.3-100	
DLI total dose until FU90				0.86
Median (*10^5 CD3+/kg)	100	100	57.5	
Range (*10^5 CD3+/kg)	0.3-1530	5-1530	0.3-900	
Age at DLI				0.71
Median (years)	52.6	52	56.7	
Range (years)	19.1-68.9	19.1-68.9	40-68.4	
Donor chimerism PB after DLI				0.1
Median – %	100	100	89	
Range – %	24-100	94-100	24-100	
Missing – no. (%)	9 (24)	8 (26)	1 (17)	
Acute GvHD after DLI1				0.45
Yes – no. (%)	8 (22)	6 (19)	2 (33)	
No – no. (%)	29 (78)	25 (81)	4 (67)	

Acute GvHD after DLI1 max grade				0.67
Grade I or II – no. (%)	5 (63)	4 (67)	1 (50)	
Grade III or IV – no. (%)	3 (37)	2 (33)	1 (50)	
Chronic GvHD after DLI1				0.35
Yes – no. (%)	4 (11)	4 (13)	0 (0)	
No – no. (%)	33 (89)	27 (87)	6 (100)	
Chronic GvHD after DLI1 max. grade				-
Limited – no. (%)	2 (50)	2 (50)	0 (0)	
Extensive – no. (%)	2 (50)	2 (50)	0 (0)	
AlloHCT2 after DLI1				0.4
Yes – no. (%)	3 (8)	2 (6)	1 (17)	
No – no. (%)	34 (92)	29 (94)	5 (83)	
CMV reactivation after DLI				0.12
Yes – no. (%)	5 (14)	3 (10)	2 (33)	
No – no. (%)	32 (86)	28 (90)	4 (67)	
Cause of death				0.41
Alive – no. (%)	17 (46)	16 (52)	1 (16.7)	
Relapse – no. (%)	9 (24)	6 (19)	3 (50)	
Infection – no. (%)	2 (5)	2 (6)	0 (0)	
Chronic GvHD – no. (%)	4 (11)	3 (10)	1 (16.7)	
Other/unknown – no. (%)	5 (14)	4 (13)	1 (16.7)	
Treatment between DLI and FU90				0.44
No additional treatment – no. (%)	10 (27)	7 (23)	3 (50)	
Only DLI – no. (%)	21 (57)	19 (61)	2 (33)	
Non-intensive therapy (+DLI) – no. (%)	2 (5)	2 (6)	1 (17)	
Intensive therapy (+DLI) – no. (%)	4 (11)	3 (10)	0 (0)	
Treatment after FU90				0.16
No – no. (%)	22 (60)	20 (65)	2 (33.3)	
DLIs only – no. (%)	3 (8)	3 (10)	0 (0)	
Non-intensive treatment – no. (%)	5 (13)	2 (6)	2 (33.3)	
Intensive treatment – no. (%)	7 (19)	6 (19)	2 (33.3)	

Abbreviations: alloHCT, allogeneic hematopoietic cell transplantation; BM, bone marrow; CMV, cytomegalovirus; CR, complete remission; CRi, CR with incomplete haematological recovery; DLI, donor lymphocyte infusion; DRI, disease risk index; FU90, follow up time point 90 days after DLI; GvHD, graft versus host disease; HCT-CI, Hematopoietic Cell Transplantation-specific Comorbidity Index; MRD, matched related donor; MMRD, mismatched related

donor; MMUD, mismatched unrelated donor; MUD, matched unrelated donor; PB, peripheral blood; pDLI, preemptive DLI; tDLI, therapeutic DLI; WBC, white blood cell.

Supplementary Table S14: Molecular characteristics at diagnosis of patients in CR/CRi at FU90.

**Mutational class at diagnosis**	**All (n=37)**	**MRD$^-$ FU90 (n=31)**	**MRD$^+$ FU90 (n=6)**	**p**
Epigenetic modifier				0.34
Mutated – no. (%)	19 (51)	17 (55)	2 (33)	
Wild type – no. (%)	18 (49)	14 (45)	4 (67)	
Nucleophosmin				0.35
Mutated – no. (%)	4 (11)	4 (13)	0 (0)	
Wild type – no. (%)	33 (89)	27 (87)	6 (100)	
Chromatin modifier				0.35
Mutated – no. (%)	4 (11)	4 (13)	0 (0)	
Wild type – no. (%)	33 (89)	27 (87)	6 (100)	
Cohesin complex				0.97
Mutated – no. (%)	6 (16)	5 (16)	1 (17)	
Wild type – no. (%)	31 (84)	26 (84)	5 (63)	
Signal transduction				0.59
Mutated – no. (%)	16 (43)	14 (45)	2 (33)	
Wild type – no. (%)	21 (57)	17 (55)	4 (67)	
Spliceosome				0.61
Mutated – no. (%)	4 (11)	3 (10)	1 (17)	
Wild type – no. (%)	33 (89)	28 (90)	5 (63)	
Myeloid Transcription factor				0.017
Mutated – no. (%)	10 (27)	6 (19)	4 (67)	
Wild type – no. (%)	27 (739	25 (81)	2 (33)	
Tumor suppressor				0.2
Mutated – no. (%)	7 (19)	7 (23)	0 (0)	
Wild type – no. (%)	30 (81)	24 (77)	6 (100)	

Supplementary Table S15: Univariate analysis of all considered variables for CIR, NRM, RFS and OS for patients in CR/CRi at FU90 (n=37). See excel file “Supplementary Table S15”.

Supplementary Table S16: Baseline characteristics of patients who were MRD$^-$ and in CR/CRi before DLI vs. a control cohort without DLI and MRD$^-$ after alloHCT.

**Characteristic**	**All (n=91)**	**no DLI (n=68)**	**DLI (n=23)**	**p**
Age at diagnosis				0.22
Median – (years)	51.7	53.7	50.7	
Range – (years)	18.3-70.8	19-70.8	18.3-65.5	
Patient sex				0.06
Male – no. (%)	51 (56)	42 (62)	9 (39)	
Female – no. (%)	40 (44)	26 (38)	14 (61)	
Diagnosis				0.63
De novo AML – no. (%)	63 (69)	48 (71)	15 (65)	
sAML / tAML / MDS/AML – no. (%)	28 (31)	20 (29)	8 (35)	
FAB–subtype				0.13
M0 + M1 + M2 – no. (%)	35 (39)	24 (35)	11 (49)	
M4 + M5 + M6 + M7 – no. (%)	32 (35)	27 (40)	5 (22)	
Not classifiable – no. (%)	24 (26)	17 (25)	7 (30)	
FLT3-ITD				0.005
Yes – no. (%)	12 (13)	5 (7)	7 (30)	
No – no. (%)	79 (87)	63 (93)	16 (70)	
Complex karyotype				0.51
Yes – no. (%)	9 (10)	6 (9)	3 (13)	
No – no. (%)	81 (89)	62 (91)	19 (83)	
Missing – no. (%)	1 (1)	0 (0)	1 (4)	
MRC Grimwade				0.38
Favorable + Intermediate – no. (%)	75 (82)	58 (85)	17 (74)	
Adverse – no. (%)	15 (17)	10 (15)	5 (22)	
Missing – no. (%)	1 (1)	0 (0)	1 (4)	
ECOG performance status at diagnosis				0.006
ECOG 0 – no. (%)	63 (69)	41 (61)	22 (96)	
ECOG 1 – no. (%)	25 (28)	24 (35)	1 (4)	
ECOG 2 – no. (%)	3 (3)	3 (4)	0 (0)	
HCT–CI at diagnosis				0.24
0-2 – no. (%)	76 (84)	55 (81)	21 (91)	
>2 – no. (%)	15 (16)	13 (19)	2 (9)	
WBC count at diagnosis				0.75
Median – (x 10^9/l)	12.3	8.30	23.9	
Range – (x 10^9/l)	0.7-223.5	0.7-223.5	1.2-115.2	
Missing – no. (%)	19 (21)	13 (19)	6 (26)	
Hemoglobin at diagnosis				0.99

Median – g/dL	9.8	9.8	9.8	
Range – g/dL	4.3-144	4.3-13.2	5-144	
Missing – no. (%)	19 (21)	13 (19)	6 (26)	
Platelet count at diagnosis				0.92
Median – (x 10^9/l)	63	57	53.5	
Range – (x 10^9/l)	7-305	11-305	7-212	
Missing – no. (%)	20 (22)	13 (19)	7 (30)	

Abbreviations: BM, bone marrow; CMV, cytomegalovirus; CR, complete remission; CRi, CR with incomplete haematological recovery; ECOG, Eastern Cooperative Oncology Group; ELN, European leukemia net; FAB, French-American-British; HCT-CI, Hematopoietic Cell Transplantation-specific Comorbidity Index; ITD, internal tandem duplication; MDS, Myelodysplastic syndrome; MRC, Medical research council; PB, peripheral blood; sAML, secondary AML; tAML, therapy-related AML; WBC, white blood cell.

Supplementary Table S17: Treatment- and transplant-associated characteristics of patients who were MRD$^-$ and in CR/CRi before DLI and a control cohort without DLI and MRD$^-$ after alloHCT.

**Characteristic**	**All (n=91)**	**no DLI (n=68)**	**DLI (n=23)**	**p**
DRI sore grouped				0.65
Low + intermediate – no. (%)	59 (65)	45 (66)	14 (39)	
High + very high – no. (%)	32 (35)	23 (34)	9 (39)	
Remission status at alloHCT1				0.47
First CR – no. (%)	52 (57)	40 (59)	12 (52)	
CRi – no. (%)	4 (4)	3 (4)	1 (4)	
Partial remission – no. (%)	4 (4)	2 (3)	2 (9)	
Second CR – no. (%)	5 (6)	5 (7)	0 (0)	
No CR/Cri/PR – no. (%)	26 (29)	18 (27)	8 (35)	
Donor match at alloHCT1				0.13
MRD – no. (%)	25 (28)	15 (22)	10 (43)	
MUD – no. (%)	44 (48)	36 (53)	8 (35)	
MMRD or MMUD – no. (%)	22 (24)	17 (25)	5 (22)	
Conditioning therapy alloHCT1				0.45

Myeloablative – no. (%)	26 (29)	18 (26)	8 (35)	
Reduced intensity – no. (%)	65 (71)	50 (74)	15 (65)	
Stem cell source at alloHCT1				0.07
PB – no. (%)	86 (95)	66 (97)	20 (87)	
BM – no. (%)	5 (5)	2 (3)	3 (13)	
Donor sex alloHCT1				0.02
Male – no. (%)	58 (64)	48 (71)	10 (43)	
Female – no. (%)	33 (36)	20 (29)	13 (57)	
Donor age alloHCT1				0.09
Median (years)	37.8	34.6	44	
Range (years)	14.9-62.6	19.5-62.6	14.9-58.7	
Missing – no. (%)	29 (32)	28 (41)	1 (4)	
Patient age alloHCT1				0.28
Median (years)	52.1	53.7	50.3	
Range (years)	18.5-70.8	19-70.8	18.5-68	
CMV status alloHCT1				0.97
Patient neg. /Donor neg. – no. (%)	33 (36)	25 (37)	8 (35)	
Any other constellation – no. (%)	57 (63)	43 (63)	14 (61)	
Missing – no. (%)	1 (1)	0 (0)	1 (4)	
Chimerism at MRD measurement				0.74
Median (years)	100	100	100	
Range (years)	77-100	97-100	77-100	
Missing – no. (%)	2 (2)	1 (1)	1 (4)	
Acute GvHD after alloHCT1 before DLI				<0.001
Yes – no. (%)	46 (51)	43 (63)	3 (13)	
No – no. (%)	45 (49)	25 (37)	20 (87)	
Acute GvHD after alloHCT1 grade				<0.001
No – no. (%)	45 (49)	25 (37)	20 (87)	
Grade I or II – no. (%)	35 (39)	32 (47)	3 (13)	
Grade III or IV – no. (%)	9 (10)	9 (13)	0 (0)	
Missing – no. (%)	2 (2)	2 (3)	0 (0)	
Chronic GvHD after alloHCT1 before DLI				<0.001
Yes – no. (%)	36 (40)	34 (50)	2 (9)	
No – no. (%)	55 (60)	34 (50)	21 (91)	
Chronic GvHD after alloHCT1 max. grade				0.002
No – no. (%)	55 (60)	34 (50)	21 (91)	
Limited – no. (%)	26 (29)	24 (35)	2 (9)	
Extensive – no. (%)	10 (11)	10 (15)	0 (0)	
Cause of death				0.006
Alive – no. (%)	72 (79)	56 (82)	16 (70)	
Relapse – no. (%)	10 (11)	4 (6)	6 (26)	
Infection – no. (%)	1 (1)	0 (0)	1 (4)	
Other/unknown – no. (%)	8 (9)	8 (12)	0 (0)	

Abbreviations: alloHCT, allogeneic hematopoietic cell transplantation; BM, bone marrow; CMV, cytomegalovirus; CR, complete remission; CRi, CR with incomplete haematological recovery; DLI, donor lymphocyte infusion; DRI, disease risk index; FU90, follow up time point 90 days after DLI; GvHD, graft versus host disease; HCT-CI, Hematopoietic Cell Transplantation-specific Comorbidity Index; MRD, matched related donor; MMRD, mismatched related donor; MMUD, mismatched unrelated donor; MUD, matched unrelated donor; PB, peripheral blood.
