# Supplementary material for: MRD as Biomarker for Response to Donor Lymphocyte Infusion after Allogeneic Hematopoietic Cell Transplantation in Patients with AML
Source: Cancers (Basel). 2023 Aug 1;15(15):3911. doi: 10.3390/cancers15153911 (PMC10416875; doi:10.3390/cancers15153911)
Supplement: Supplementary file 1 [file cancers-15-03911-s001.zip › Supplementary Table S11.pdf]

Supplementary Table S11: Variables for univariate analysis in 34 patients who are in CR/CRi at FU30

Variables	N patients	First category (name)	Second category (name)	First category (n)	Second category (n)	Cumulative incidence of relapse<br>HR#	95% CI (lower limit)	95% CI (upper limit)	P	5 year CIR (%) category 1	5 year CIR (%) category 2	Selected for multivariate analysis
pre DLI median Age	34	Age 54 years or younger	Age above 54 years	20	14	0.43	0.12	1.48	0.181	40	14	
Patient sex	34	Female	Male	16	18	0.49	0.14	1.64	0.245	38	22	
Diagnosis	34	De novo AML	sAML / tAML / MDS	21	13	1.02	0.28	3.72	0.971	29	31	
Extramedullary manifestation at diagnosis	34	No	Yes	30	4	0.87	0.09	8.31	0.900	30	NA	
FAB subtype	23	M0 + M1 + M2	M4 + M5 + M6 + M7	18	5	1.28	0.23	7.08	0.775	33	40	
FLT3-ITD status at diagnosis	34	No	Yes	27	7	0.38	0.05	2.92	0.350	33	14	
Complex karyotype	34	No	Yes	29	5	3.43	0.87	13.56	0.078	24	NA	1
2022 ELN risk group	31	Favorable + Intermediate	Adverse	15	16	0.76	0.24	2.41	0.641	33	31	
MRC Grimwade	34	Favorable + Intermediate	Adverse	25	9	2.09	0.61	7.20	0.242	24	44	
ECOG performance status at diagnosis	34	ECOG 0	ECOG 1	32	2	1.35	0.34	5.39	0.675	31	0	*
HCT–CI at diagnosis	34	0-2	>2	30	4	1.69	0.41	6.91	0.463	30	25	

WBC count at diagnosis	25	Median or less	Above median	13	12	0.57	0.15	2.17	0.408	38	25	
Hemoglobin at diagnosis	25	Median or less	Above median	12	13	1.29	0.37	4.56	0.687	25	38	
Platelet count at diagnosis	25	Median or less	Above median	16	9	0.84	0.22	3.16	0.799	38	22	
Number of chemotherapy cycles before alloHCT1	34	0-1 cycles	≥2	9	25	1.87	0.42	8.27	0.409	22	32	
Donor match at alloHCT1	34	MRD	MUD	13	14	0.98	0.25	3.89	0.970	31	29	
Donor match at alloHCT1	34	MRD	MMRD or MMUD	13	7	1.37	0.34	5.52	0.660	31	29	
Conditioning therapy alloHCT1	34	Myeloablative	Reduced intensity	9	25	1.62	0.37	7.17	0.526	22	32	
Stem cell source at alloHCT1	34	PB	BM	27	7	1.51	0.44	5.23	0.512	30	29	
Donor sex alloHCT1	34	Female	Male	13	21	1.09	0.33	3.58	0.881	31	29	
Donor age alloHCT1	33	Age 36 years or younger	Age above 36 years	14	19	0.96	0.31	2.99	0.948	29	32	
CMV status alloHCT1	34	Patient neg. /Donor neg.	Any other constellation	12	22	2.87	0.62	13.24	0.178	17	36	
Epigenetic modifier pre DLI	34	MRD-	MRD+	25	9	1.75	0.54	5.67	0.352	28	33	
Nucleophosmin pre DLI	34	MRD-	MRD+	33	1	0.00	0.00	0.00	0.000	30	NA	*
Cohesin complex pre DLI	34	MRD-	MRD+	32	2	5.84	2.26	15.10	0.000	25	NA	*

Signal transduction pre DLI	34	MRD-	MRD+	32	2	0.00	0.00	0.00	0.000	31	NA	*
Spliceosome pre DLI	34	MRD-	MRD+	31	3	0.97	0.16	5.74	0.973	29	NA	*
Myeloid Transcription factor pre DLI	34	MRD-	MRD+	30	4	0.66	0.11	4.02	0.652	30	25	
Tumor suppressor pre DLI	34	MRD-	MRD+	32	2	0.00	0.00	0.00	0.000	31	0	*
DRI Score grouped	34	Low + intermediate	High + very high	20	14	1.31	0.40	4.28	0.653	25	36	
FU30 MRD status	34	Negative	Positive	25	9	2.64	0.86	8.13	0.090	24	44	2

Number of selected variables for multivariate analysis	2

						Non relapse mortality						
**Variables**	**N patients**	**First category (name)**	**Second category (name)**	**First category (n)**	**Second category (n)**	**HR#**	**95% CI (lower limit)**	**95% CI (upper limit)**	**P**	**5 year NRM (%) category 1**	**5 year NRM (%) category 2**	**Selected for multivariate analysis**
pre DLI median Age	34	Age 54 years or younger	Age above 54 years	20	14	3.78	0.76	18.9	0.105	10	29	1
Patient sex	34	Female	Male	16	18	1.14	0.26	4.91	0.865	20	17	
Diagnosis	34	De novo AML	sAML / tAML / MDS	21	13	1.19	0.29	4.93	0.815	19	15	

Extramedullary manifestation at diagnosis	34	No	Yes	30	4	5.21	0.89	30.4	0.066	14	NA	2
FAB subtype	23	M0 + M1 + M2	M4 + M5 + M6 + M7	18	5	1.16	0.14	9.73	0.890	17	20	
FLT3-ITD status at diagnosis	34	No	Yes	27	7	1.71	0.35	8.25	0.504	15	29	
Complex karyotype	34	No	Yes	29	5	1.09	0.14	8.78	0.935	17	NA	
2022 ELN risk group	31	Favorable + Intermediate	Adverse	15	16	2.78	0.61	12.8	0.188	13	25	
MRC Grimwade	34	Favorable + Intermediate	Adverse	25	9	1.27	0.26	6.16	0.767	16	22	
ECOG performance status at diagnosis	34	ECOG 0	ECOG 1	32	2	0.00	0.00	0.00	0.000	19	0	*
HCT–CI at diagnosis	34	0-2	>2	30	4	1.34	0.15	12.2	0.796	17	25	
WBC count at diagnosis	25	Median or less	Above median	13	12	1.96	0.38	10.2	0.425	8	25	
Hemoglobin at diagnosis	25	Median or less	Above median	12	13	0.58	0.10	3.39	0.548	17	15	
Platelet count at diagnosis	25	Median or less	Above median	16	9	1.26	0.22	7.33	0.796	12	22	
Number of chemotherapy cycles before alloHCT1	34	0-1 cycles	≥2	9	25	0.45	0.11	1.85	0.269	22	16	
Donor match at alloHCT1	34	MRD	MUD	13	14	2.53	0.51	12.6	0.260	15	30	

Donor match at alloHCT1	34	MRD	MMRD or MMUD	13	7	0.00	0.00	0.00	0.000	15	30	*
Conditioning therapy alloHCT1	34	Myeloablative	Reduced intensity	9	25	2.02	0.23	17.7	0.525	11	20	
Stem cell source at alloHCT1	34	PB	BM	27	7	0.59	0.08	4.54	0.613	19	14	
Donor sex alloHCT1	34	Female	Male	13	21	1.41	0.27	7.33	0.685	15	19	
Donor age alloHCT1	33	Age 36 years or younger	Age above 36 years	14	19	0.28	0.05	1.50	0.138	30	11	
CMV status alloHCT1	34	Patient neg. /Donor neg.	Any other constellation	12	22	1.52	0.32	7.27	0.599	8	24	
Epigenetic modifier pre DLI	34	MRD-	MRD+	25	9	2.21	0.53	9.19	0.275	12	33	
Nucleophosmin pre DLI	34	MRD-	MRD+	33	1	5.96	2.53	14.0	0.000	15	NA	*
Cohesin complex pre DLI	34	MRD-	MRD+	32	2	0.00	0.00	0.00	0.000	19	NA	*
Signal transduction pre DLI	34	MRD-	MRD+	32	2	9.09	3.14	26.3	0.000	12	NA	*
Spliceosome pre DLI	34	MRD-	MRD+	31	3	1.98	0.27	14.6	0.501	17	NA	*
Myeloid Transcription factor pre DLI	34	MRD-	MRD+	30	4	0.00	0.00	0.00	0.000	20	0	*
Tumor suppressor pre DLI	34	MRD-	MRD+	32	2	8.09	2.71	24.2	0.000	16	50	*
DRI Score grouped	34	Low + intermediate	High + very high	20	14	2.02	0.48	8.50	0.338	15	21	

FU30 MRD status	34	Negative	Positive	25	9	1.04	0.22	4.88	0.961	21	11	

Number of selected variables for multivariate analysis	2

						Relapse free survival						
**Variables**	**N patients**	**First category (name)**	**Second category (name)**	**First category (n)**	**Second category (n)**	**HR#**	**95% CI (lower limit)**	**95% CI (upper limit)**	**P**	**5 year RFS (%) category 1**	**5 year RFS (%) category 2**	**Selected for multivariate analysis**
pre DLI median Age	34	Age 54 years or younger	Age above 54 years	20	14	1.03	0.40	2.61	0.956	50	56	
Patient sex	34	Female	Male	16	18	0.66	0.26	1.68	0.387	43	61	
Diagnosis	34	De novo AML	sAML / tAML / MDS	21	13	1.18	0.45	3.06	0.738	52	54	
Extramedullary manifestation at diagnosis	34	No	Yes	30	4	4.45	1.25	15.91	0.021	56	NA	
FAB subtype	23	M0 + M1 + M2	M4 + M5 + M6 + M7	18	5	1.38	0.38	5.04	0.626	49	40	
FLT3-ITD status at diagnosis	34	No	Yes	27	7	0.78	0.22	2.72	0.696	52	57	
Complex karyotype	34	No	Yes	29	5	3.47	1.08	11.16	0.037	58	NA	1
2022 ELN risk group	31	Favorable + Intermediate	Adverse	15	16	1.47	0.58	3.74	0.417	53	44	
MRC Grimwade	34	Favorable + Intermediate	Adverse	25	9	2.26	0.83	6.18	0.111	60	33	

ECOG performance status at diagnosis	34	ECOG 0	ECOG 1	32	2	0.63	0.08	4.79	0.659	50	100	*
HCT–CI at diagnosis	34	0-2	>2	30	4	1.76	0.50	6.18	0.374	53	50	
WBC count at diagnosis	25	Median or less	Above median	13	12	1.03	0.35	3.03	0.951	54	50	
Hemoglobin at diagnosis	25	Median or less	Above median	12	13	1.02	0.36	2.93	0.970	58	46	
Platelet count at diagnosis	25	Median or less	Above median	16	9	1.03	0.34	3.10	0.954	50	56	
Number of chemotherapy cycles before alloHCT1	34	0-1 cycles	≥2	9	25	0.96	0.34	2.71	0.945	56	52	
Donor match at alloHCT1	34	MRD	MUD	13	14	1.70	0.60	4.84	0.318	54	42	
Donor match at alloHCT1	34	MRD	MMRD or MMUD	13	7	0.80	0.20	3.19	0.747	54	42	
Conditioning therapy alloHCT1	34	Myeloablative	Reduced intensity	9	25	1.86	0.54	6.43	0.330	67	48	
Stem cell source at alloHCT1	34	PB	BM	27	7	1.03	0.34	3.15	0.954	51	57	
Donor sex alloHCT1	34	Female	Male	13	21	1.19	0.44	3.17	0.731	54	52	
Donor age alloHCT1	33	Age 36 years or younger	Age above 36 years	14	19	0.57	0.22	1.44	0.235	42	58	
CMV status alloHCT1	34	Patient neg. /Donor neg.	Any other constellation	12	22	2.57	0.84	7.87	0.098	75	40	2
Epigenetic modifier pre DLI	34	MRD-	MRD+	25	9	2.21	0.85	5.72	0.102	60	33	

Nucleophosmin pre DLI	34	MRD-	MRD+	33	1	1.63	0.21	12.37	0.637	55	NA	*
Cohesin complex pre DLI	34	MRD-	MRD+	32	2	2.82	0.63	12.67	0.176	56	NA	*
Signal transduction pre DLI	34	MRD-	MRD+	32	2	2.54	0.57	11.21	0.220	56	NA	*
Spliceosome pre DLI	34	MRD-	MRD+	31	3	1.52	0.34	6.71	0.581	54	NA	*
Myeloid Transcription factor pre DLI	34	MRD-	MRD+	30	4	0.35	0.05	2.63	0.306	50	75	
Tumor suppressor pre DLI	34	MRD-	MRD+	32	2	2.50	0.57	11.01	0.224	53	50	*
DRI Score grouped	34	Low + intermediate	High + very high	20	14	1.86	0.74	4.71	0.189	60	43	
FU30 MRD status	34	Negative	Positive	25	9	2.00	0.77	5.17	0.154	55	44	3

Number of selected variables for multivariate analysis	3

						Overall survival						
**Variables**	**N patients**	**First category (name)**	**Second category (name)**	**First category (n)**	**Second category (n)**	**HR#**	**95% CI (lower limit)**	**95% CI (upper limit)**	**P**	**5 year OS (%) category 1**	**5 year OS (%) category 2**	**Selected for multivariate analysis**

pre DLI median Age	34	Age 54 years or younger	Age above 54 years	20	14	0.86	0.31	2.37	0.769	46	62	
Patient sex	34	Female	Male	16	18	0.83	0.31	2.22	0.713	45	60	
Diagnosis	34	De novo AML	sAML / tAML / MDS	21	13	1.34	0.50	3.63	0.559	53	53	
Extramedullary manifestation at diagnosis	34	No	Yes	30	4	5.10	1.39	18.67	0.014	57	NA	1
FAB subtype	23	M0 + M1 + M2	M4 + M5 + M6 + M7	18	5	1.40	0.37	5.28	0.621	49	40	
FLT3-ITD status at diagnosis	34	No	Yes	27	7	0.83	0.24	2.92	0.771	53	48	
Complex karyotype	34	No	Yes	29	5	4.91	1.36	17.68	0.015	60	NA	2
2022 ELN risk group	31	Favorable + Intermediate	Adverse	15	16	2.02	0.73	5.58	0.175	57	40	
MRC Grimwade	34	Favorable + Intermediate	Adverse	25	9	2.87	1.00	8.26	0.050	62	22	3
ECOG performance status at diagnosis	34	ECOG 0	ECOG 1	32	2	0.00	0.00	0.00	0.998	50	100	*
HCT–CI at diagnosis	34	0-2	>2	30	4	1.25	0.28	5.51	0.771	53	50	
WBC count at diagnosis	25	Median or less	Above median	13	12	1.30	0.42	4.04	0.654	58	49	
Hemoglobin at diagnosis	25	Median or less	Above median	12	13	1.40	0.44	4.41	0.569	64	44	
Platelet count at diagnosis	25	Median or less	Above median	16	9	0.97	0.29	3.24	0.963	51	56	

Number of chemotherapy cycles before alloHCT1	34	0-1 cycles	≥2	9	25	1.11	0.36	3.44	0.860	62	50	
Donor match at alloHCT1	34	MRD	MUD	13	14	1.63	0.56	4.71	0.370	50	48	
Donor match at alloHCT1	34	MRD	MMRD or MMUD	13	7	0.58	0.12	2.90	0.510	50	48	
Conditioning therapy alloHCT1	34	Myeloablative	Reduced intensity	9	25	1.74	0.49	6.12	0.388	59	50	
Stem cell source at alloHCT1	34	PB	BM	27	7	0.71	0.20	2.50	0.596	53	51	
Donor sex alloHCT1	34	Female	Male	13	21	1.01	0.37	2.78	0.985	47	55	
Donor age alloHCT1	33	Age 36 years or younger	Age above 36 years	14	19	0.68	0.25	1.80	0.434	45	56	
CMV status alloHCT1	34	Patient neg. /Donor neg.	Any other constellation	12	22	3.19	0.90	11.29	0.072	83	35	4
Epigenetic modifier pre DLI	34	MRD-	MRD+	25	9	1.94	0.70	5.35	0.202	61	33	
Nucleophosmin pre DLI	34	MRD-	MRD+	33	1	2.39	0.31	18.58	0.404	55	NA	*
Cohesin complex pre DLI	34	MRD-	MRD+	32	2	1.21	0.16	9.32	0.855	54	NA	*
Signal transduction pre DLI	34	MRD-	MRD+	32	2	3.85	0.84	17.70	0.083	57	NA	*
Spliceosome pre DLI	34	MRD-	MRD+	31	3	0.89	0.12	6.75	0.906	52	NA	*
Myeloid Transcription factor pre DLI	34	MRD-	MRD+	30	4	0.50	0.07	3.82	0.505	52	67	

Tumor suppressor pre DLI	34	MRD-	MRD+	32	2	2.66	0.60	11.75	0.198	53	50	*
DRI Score grouped	34	Low + intermediate	High + very high	20	14	2.00	0.75	5.38	0.168	57	50	
FU30 MRD status	34	Negative	Positive	25	9	1.20	0.42	3.46	0.737	54	52	

Number of selected variables for multivariate analysis	4

* not considered due to lack of any events or too few patients in one of the categories

#Hazard ratios greater than or less than 1 indicate an increased or decreased risk, respectively, of an event for the second category listed.

Abbreviations: CI, confidence interval; CMV, cytomegalovirus; CR, complete remission; ECOG, Eastern Cooperative Group performance status; ELN, European LeukemiaNet; HCT, hematopoietic cell transplantation; HCT-CI, hematopoietic cell transplantation comorbidity index; HR, hazard ratio; ITD, internal tandem duplication; MMRD, mismatched related donor; MMUD, mismatched unrelated donor; MRDonor, matched related donor; MUD, matched unrelated donor; MVA, multivariate analysis; MRD, measurable residual disease; P, P value; WBC, white blood cell count.
