# Supplementary material for: MRD as Biomarker for Response to Donor Lymphocyte Infusion after Allogeneic Hematopoietic Cell Transplantation in Patients with AML
Source: Cancers (Basel). 2023 Aug 1;15(15):3911. doi: 10.3390/cancers15153911 (PMC10416875; doi:10.3390/cancers15153911)
Supplement: Supplementary file 1 [file cancers-15-03911-s001.zip › Supplementary Table S15.pdf]

Supplementary Table S15: Variables for univariate analysis in 37 patients who are in CR/CRi at FU90.

						Cumulative incidence of relapse						
Variables	N patients	First category (name)	Second category (name)	First category (n)	Second category (n)	HR#	95% CI (lower limit)	95% CI (upper limit)	P	5 year CIR (%) category 1	5 year CIR (%) category 2	Selected for multivariate analysis
pre DLI median Age	37	Age 54 years or younger	Age above 54 years	21	16	0.55	0.17	1.74	0.312	39	19	
Patient sex	37	Female	Male	19	18	0.50	0.15	1.63	0.247	37	22	
Diagnosis	37	De novo AML	sAML / tAML / MDS/AML	23	14	1.38	0.42	4.56	0.598	26	36	
Extramedullary manifestation at diagnosis	37	No	Yes	32	5	0.62	0.07	5.70	0.673	31	NA	
FAB subtype	23	M0 + M1 + M2	M4 + M5 + M6 + M7	16	7	0.69	0.13	3.69	0.667	38	29	
FLT3-ITD status at diagnosis	37	No	Yes	29	8	0.30	0.04	2.32	0.251	35	12	
Complex karyotype	37	No	Yes	32	5	3.22	0.85	12.25	0.086	25	NA	1
2022 ELN risk group	34	Favorable + Intermediate	Adverse	19	15	1.07	0.33	3.40	0.912	26	33	
MRC Grimwade	37	Favorable + Intermediate	Adverse	27	10	2.68	0.84	8.53	0.095	22	50	2
ECOG performance status at diagnosis	37	ECOG 0	ECOG 1	35	2	1.29	0.34	4.87	0.709	32	0	*
HCT–CI at diagnosis	37	0-2	>2	32	5	2.54	0.70	9.26	0.156	28	40	
WBC count at diagnosis	27	Median or less	Above median	16	11	0.66	0.18	2.46	0.532	38	27	

Hemoglobin at diagnosis	27	Median or less	Above median	13	14	1.69	0.52	5.51	0.388	23	43	
Platelet count at diagnosis	27	Median or less	Above median	15	12	0.45	0.12	1.64	0.224	47	17	
Number of chemotherapy cycles before alloHCT1	37	0-1 cycles	≥2	9	28	1.83	0.43	7.83	0.416	22	32	
Donor match at alloHCT1	37	MRD	MUD	13	16	1.10	0.30	4.03	0.890	31	31	
Donor match at alloHCT1	37	MRD	MMRD or MMUD	13	8	1.14	0.29	4.53	0.850	31	31	
Conditioning therapy alloHCT1	37	Myeloablative	Reduced intensity	10	27	1.85	0.43	8.01	0.413	20	33	
Stem cell source at alloHCT1	37	PB	BM	29	8	2.04	0.65	6.38	0.221	28	38	
Donor sex alloHCT1	37	Female	Male	16	21	1.05	0.34	3.18	0.936	31	29	
Donor age alloHCT1	36	Age 36 years or younger	Age above 36 years	17	19	0.91	0.30	2.72	0.861	30	32	
CMV status alloHCT1	37	Patient neg. /Donor neg.	Any other constellation	13	24	1.75	0.46	6.58	0.409	23	34	
Epigenetic modifier pre DLI	37	MRD-	MRD+	26	11	1.17	0.37	3.70	0.790	31	27	
Nucleophosmin preDLI	37	MRD-	MRD+	33	4	0.00	0.00	0.00	0.000	34	NA	*
Cohesin complex preDLI	37	MRD-	MRD+	35	2	5.33	2.24	12.71	0.000	26	NA	*
Signal transduction preDLI	37	MRD-	MRD+	33	4	0.00	0.00	0.00	0.000	34	NA	*

Spliceosome preDLI	37	MRD-	MRD+	34	3	0.95	0.16	5.56	0.957	30	NA	*
Myeloid Transcription factor preDLI	37	MRD-	MRD+	31	6	0.39	0.06	2.51	0.319	32	17	
Tumor suppressor preDLI	37	MRD-	MRD+	34	3	0.00	0.00	0.00	0.000	33	0	*
DRI Score grouped	37	Low + intermediate	High + very high	23	14	1.96	0.63	6.04	0.243	22	43	
FU90 MRD status	37	Negative	Positive	31	6	3.02	1.02	8.94	0.047	23	NA	3

Number of selected variables for multivariate analysis	3

						Non relapse mortality						
**Variables**	**N patients**	**First category (name)**	**Second category (name)**	**First category (n)**	**Second category (n)**	**HR#**	**95% CI (lower limit)**	**95% CI (upper limit)**	**P**	**5 year NRM (%) category 1**	**5 year NRM (%) category 2**	**Selected for multivariate analysis**
pre DLI median Age	37	Age 54 years or younger	Age above 54 years	21	16	5.79	1.24	27.1	0.026	10	46	1
Patient sex	37	Female	Male	19	18	2.57	0.68	9.77	0.166	18	35	
Diagnosis	37	De novo AML	sAML / tAML / MDS/AML	23	14	1.56	0.48	5.05	0.462	23	31	
Extramedullary manifestation at diagnosis	37	No	Yes	32	5	4.78	1.10	20.9	0.037	22	NA	2
FAB subtype	23	M0 + M1 + M2	M4 + M5 + M6 + M7	16	7	4.15	0.76	22.8	0.101	13	43	
FLT3-ITD status at diagnosis	37	No	Yes	29	8	1.47	0.43	5.07	0.540	22	41	

Complex karyotype	37	No	Yes	32	5	0.82	0.10	6.74	0.853	27	NA		
2022 ELN risk group	34	Favorable + Intermediate	Adverse	19	15	1.31	0.40	4.30	0.652	30	29		
MRC Grimwade	37	Favorable + Intermediate	Adverse	27	10	0.76	0.16	3.58	0.731	28	20		
ECOG performance status at diagnosis	37	ECOG 0	ECOG 1	35	2	0.00	0.00	0.00	0.000	28	0	*	
HCT–CI at diagnosis	37	0-2	>2	32	5	0.68	0.07	6.19	0.731	28	20		
WBC count at diagnosis	27	Median or less	Above median	16	11	1.73	0.38	7.94	0.483	16	27		
Hemoglobin at diagnosis	27	Median or less	Above median	13	14	0.93	0.20	4.36	0.924	15	25		
Platelet count at diagnosis	27	Median or less	Above median	15	12	1.31	0.28	6.02	0.729	13	28		
Number of chemotherapy cycles before alloHCT1	37	0-1 cycles	≥2	9	28	0.67	0.19	2.40	0.537	22	27		
Donor match at alloHCT1	37	MRD	MUD	13	16	1.58	0.39	6.44	0.520	23	35		
Donor match at alloHCT1	37	MRD	MMRD or MMUD	13	8	0.41	0.05	3.56	0.420	23	35		
Conditioning therapy alloHCT1	37	Myeloablative	Reduced intensity	10	27	2.95	0.35	24.7	0.319	10	31		
Stem cell source at alloHCT1	37	PB	BM	29	8	0.34	0.04	2.64	0.303	31	12		
Donor sex alloHCT1	37	Female	Male	16	21	3.01	0.65	14.1	0.161	12	35		
Donor age alloHCT1	36	Age 36 years or younger	Age above 36 years	17	19	0.33	0.08	1.38	0.129	44	16		

CMV status alloHCT1	37	Patient neg. /Donor neg.	Any other constellation	13	24	2.66	0.59	11.9	0.200	8	39	
Epigenetic modifier pre DLI	37	MRD-	MRD+	26	11	2.51	0.77	8.20	0.128	17	45	
Nucleophosmin preDLI	37	MRD-	MRD+	33	4	8.91	2.46	32.3	0.001	20	NA	*
Cohesin complex preDLI	37	MRD-	MRD+	35	2	0.00	0.00	0.00	0.000	28	NA	*
Signal transduction preDLI	37	MRD-	MRD+	33	4	7.72	2.13	27.9	0.002	20	NA	*
Spliceosome preDLI	37	MRD-	MRD+	34	3	1.42	0.17	11.6	0.746	26	NA	*
Myeloid Transcription factor preDLI	37	MRD-	MRD+	31	6	1.25	0.32	4.87	0.751	23	52	
Tumor suppressor preDLI	37	MRD-	MRD+	34	3	3.82	1.24	11.8	0.020	26	33	
DRI Score grouped	37	Low + intermediate	High + very high	23	14	1.11	0.32	3.82	0.866	30	21	
FU90 MRD status	37	Negative	Positive	31	6	1.13	0.30	4.27	0.860	24	NA	

Number of selected variables for multivariate analysis	2

Relapse free survival

Variables	N patients	First category (name)	Second category (name)	First category (n)	Second category (n)	HR#	95% CI (lower limit)	95% CI (upper limit)	P	5 year RFS (%) category 1	5 year RFS (%) category 2	Selected for multivariate analysis
pre DLI median Age	37	Age 54 years or younger	Age above 54 years	21	16	1.54	0.67	3.58	0.312	52	35	
Patient sex	37	Female	Male	19	18	1.09	0.47	2.51	0.848	45	42	
Diagnosis	37	De novo AML	sAML / tAML / MDS/AML	23	14	1.64	0.70	3.81	0.253	51	33	
Extramedullary manifestation at diagnosis	37	No	Yes	32	5	3.59	1.19	10.86	0.024	47	NA	1
FAB subtype	23	M0 + M1 + M2	M4 + M5 + M6 + M7	16	7	2.05	0.68	6.18	0.204	49	29	
FLT3-ITD status at diagnosis	37	No	Yes	29	8	0.66	0.22	1.97	0.455	43	47	
Complex karyotype	37	No	Yes	32	5	3.17	1.02	9.90	0.047	48	NA	2
2022 ELN risk group	34	Favorable + Intermediate	Adverse	19	15	1.29	0.55	3.06	0.557	44	37	
MRC Grimwade	37	Favorable + Intermediate	Adverse	27	10	2.24	0.89	5.62	0.087	50	30	
ECOG performance status at diagnosis	37	ECOG 0	ECOG 1	35	2	0.47	0.06	3.56	0.467	40	100	*
HCT–CI at diagnosis	37	0-2	>2	32	5	1.75	0.58	5.29	0.323	44	40	
WBC count at diagnosis	27	Median or less	Above median	16	11	1.15	0.41	3.24	0.792	46	45	
Hemoglobin at diagnosis	27	Median or less	Above median	13	14	1.52	0.56	4.10	0.408	62	32	

Platelet count at diagnosis	27	Median or less	Above median	15	12	0.62	0.22	1.73	0.360	40	56	
Number of chemotherapy cycles before alloHCT1	37	0-1 cycles	≥2	9	28	1.11	0.41	3.02	0.834	56	41	
Donor match at alloHCT1	37	MRD	MUD	13	16	1.40	0.53	3.67	0.497	46	33	
Donor match at alloHCT1	37	MRD	MMRD or MMUD	13	8	0.64	0.19	2.20	0.477	46	33	
Conditioning therapy alloHCT1	37	Myeloablative	Reduced intensity	10	27	2.40	0.71	8.16	0.161	70	36	
Stem cell source at alloHCT1	37	PB	BM	29	8	1.01	0.37	2.74	0.988	41	50	
Donor sex alloHCT1	37	Female	Male	16	21	1.71	0.70	4.21	0.240	56	37	
Donor age alloHCT1	36	Age 36 years or younger	Age above 36 years	17	19	0.53	0.22	1.26	0.153	26	53	
CMV status alloHCT1	37	Patient neg. /Donor neg.	Any other constellation	13	24	2.61	0.95	7.19	0.064	69	27	3
Epigenetic modifier pre DLI	37	MRD-	MRD+	26	11	1.84	0.78	4.32	0.161	52	27	
Nucleophosmin preDLI	37	MRD-	MRD+	33	4	1.95	0.56	6.75	0.292	47	NA	
Cohesin complex preDLI	37	MRD-	MRD+	35	2	2.62	0.59	11.57	0.204	46	NA	
Signal transduction preDLI	37	MRD-	MRD+	33	4	1.74	0.50	6.03	0.384	47	NA	
Spliceosome preDLI	37	MRD-	MRD+	34	3	1.27	0.29	5.49	0.748	44	NA	

Myeloid Transcription factor preDLI	37	MRD-	MRD+	31	6	0.60	0.18	2.03	0.410	44	31	
Tumor suppressor preDLI	37	MRD-	MRD+	34	3	1.23	0.29	5.27	0.785	42	67	
DRI Score grouped	37	Low + intermediate	High + very high	23	14	1.90	0.81	4.43	0.138	48	36	
FU90 MRD status	37	Negative	Positive	31	6	2.27	0.87	5.92	0.095	54	NA	4

Number of selected variables for multivariate analysis	4

						Overall survival						
**Variables**	**N patients**	**First category (name)**	**Second category (name)**	**First category (n)**	**Second category (n)**	**HR#**	**95% CI (lower limit)**	**95% CI (upper limit)**	**P**	**5 year OS (%) category 1**	**5 year OS (%) category 2**	**Selected for multivariate analysis**
pre DLI median Age	37	Age 54 years or younger	Age above 54 years	21	16	1.46	0.61	3.50	0.401	45	39	
Patient sex	37	Female	Male	19	18	1.33	0.55	3.21	0.528	45	40	
Diagnosis	37	De novo AML	sAML / tAML / MDS/AML	23	14	1.85	0.77	4.46	0.171	51	33	
Extramedullary manifestation at diagnosis	37	No	Yes	32	5	3.91	1.27	12.03	0.017	46	NA	1
FAB subtype	23	M0 + M1 + M2	M4 + M5 + M6 + M7	16	7	2.17	0.68	6.89	0.188	48	29	
FLT3-ITD status at diagnosis	37	No	Yes	29	8	0.67	0.22	2.00	0.470	43	40	

Complex karyotype	37	No	Yes	32	5	4.19	1.25	14.07	0.021	48	NA	2
2022 ELN risk group	34	Favorable + Intermediate	Adverse	19	15	1.60	0.65	3.96	0.306	45	34	
MRC Grimwade	37	Favorable + Intermediate	Adverse	27	10	2.64	1.02	6.82	0.045	51	20	3
ECOG performance status at diagnosis	37	ECOG 0	ECOG 1	35	2	0.00	0.00	NA	0.998	39	100	*
HCT–CI at diagnosis	37	0-2	>2	32	5	1.41	0.41	4.87	0.583	42	40	
WBC count at diagnosis	27	Median or less	Above median	16	11	1.25	0.43	3.62	0.679	48	44	
Hemoglobin at diagnosis	27	Median or less	Above median	13	14	2.09	0.70	6.23	0.188	66	29	
Platelet count at diagnosis	27	Median or less	Above median	15	12	0.65	0.22	1.94	0.439	39	56	
Number of chemotherapy cycles before alloHCT1	37	0-1 cycles	≥2	9	28	1.32	0.44	3.96	0.619	58	39	
Donor match at alloHCT1	37	MRD	MUD	13	16	1.42	0.54	3.74	0.483	37	39	
Donor match at alloHCT1	37	MRD	MMRD or MMUD	13	8	0.55	0.14	2.12	0.383	37	39	
Conditioning therapy alloHCT1	37	Myeloablative	Reduced intensity	10	27	2.33	0.68	7.97	0.178	54	38	
Stem cell source at alloHCT1	37	PB	BM	29	8	0.73	0.24	2.18	0.570	43	45	
Donor sex alloHCT1	37	Female	Male	16	21	1.49	0.59	3.74	0.396	47	39	
Donor age alloHCT1	36	Age 36 years or younger	Age above 36 years	17	19	0.59	0.24	1.44	0.248	27	51	

CMV status alloHCT1	37	Patient neg. /Donor neg.	Any other constellation	13	24	3.01	0.99	9.11	0.052	77	23	4
Epigenetic modifier pre DLI	37	MRD-	MRD+	26	11	1.66	0.68	4.07	0.268	51	27	
Nucleophosmin preDLI	37	MRD-	MRD+	33	4	2.48	0.69	8.91	0.164	46	NA	
Cohesin complex preDLI	37	MRD-	MRD+	35	2	1.01	0.13	7.66	0.992	44	NA	*
Signal transduction preDLI	37	MRD-	MRD+	33	4	2.37	0.66	8.50	0.187	46	NA	
Spliceosome preDLI	37	MRD-	MRD+	34	3	0.71	0.09	5.29	0.734	42	NA	*
Myeloid Transcription factor preDLI	37	MRD-	MRD+	31	6	0.83	0.24	2.84	0.762	45	27	
Tumor suppressor preDLI	37	MRD-	MRD+	34	3	1.38	0.32	5.96	0.667	41	67	*
DRI Score grouped	37	Low + intermediate	High + very high	23	14	2.03	0.84	4.94	0.118	44	43	
FU90 MRD status	37	Negative	Positive	31	6	1.86	0.67	5.18	0.236	52	NA	

Number of selected variables for multivariate analysis	4

* not considered due to lack of any events or too few patients in one of the categories

#Hazard ratios greater than or less than 1 indicate an increased or decreased risk, respectively, of an event for the second category listed.

Abbreviations: CI, confidence interval; CMV, cytomegalovirus; CR, complete remission; ECOG, Eastern Cooperative Group performance status; ELN, European LeukemiaNet; HCT, hematopoietic cell transplantation; HCT-CI, hematopoietic cell transplantation comorbidity index; HR, hazard ratio; ITD, internal tandem duplication; MMRD, mismatched related donor; MMUD, mismatched unrelated donor; MRDonor, matched related donor; MUD, matched unrelated donor; MRD, measurable residual disease; MVA, multivariate Analysis; P, P value; WBC, white blood cell count.
