# Supplementary material for: MRD as Biomarker for Response to Donor Lymphocyte Infusion after Allogeneic Hematopoietic Cell Transplantation in Patients with AML
Source: Cancers (Basel). 2023 Aug 1;15(15):3911. doi: 10.3390/cancers15153911 (PMC10416875; doi:10.3390/cancers15153911)
Supplement: Supplementary file 1 [file cancers-15-03911-s001.zip › Supplementary Table S7.pdf]

Supplementary Table S7: Univariate analysis variables for 76 patient cohort (all)

						Event free survival						
**Variables**	**N patients**	**First category (name)**	**Second category (name)**	**First category (n)**	**Second category (n)**	**HR#**	**95% CI (lower limit)**	**95% CI (upper limit)**	**P**	**5 year CIR (%) category 1**	**5 year CIR (%) category 2**	**Selected for multivariate analysis**
pre DLI median Age	76	Age 54 years or younger	Age above 54 years	38	38	1.55	0.89	2.69	0.121	43	26	
Patient sex	76	Female	Male	34	42	1.10	0.63	1.90	0.744	38	31	
Diagnosis	76	De novo AML	sAML / tAML / MDS/AML	44	32	1.07	0.62	1.86	0.804	36	32	
Extramedullary manifestation at diagnosis	76	No	Yes	68	8	2.48	1.11	5.57	0.028	37	NA	1
FAB subtype	51	M0 + M1 + M2	M4 + M5 + M6 + M7	29	22	1.76	0.91	3.40	0.094	38	24	2
FLT3-ITD status at diagnosis	75	No	Yes	59	16	0.74	0.37	1.49	0.403	34	36	
Complex karyotype	75	No	Yes	64	11	2.66	1.30	5.44	0.007	39	NA	3
2017 ELN risk group	70	Favorable + Intermediate	Adverse	34	36	1.54	0.88	2.71	0.134	40	25	
MRC Grimwade	75	Favorable + Intermediate	Adverse	56	19	2.06	1.12	3.77	0.020	41	16	4
ECOG performance status at diagnosis	76	ECOG 0	ECOG 1	70	6	1.82	0.72	4.60	0.204	34	33	
HCT–CI at diagnosis	76	0-2	>2	67	9	1.30	0.58	2.89	0.525	34	33	

WBC count at diagnosis	50	Median or less	Above median	24	26	1.11	0.55	2.22	0.774	40	40	
Hemoglobin at diagnosis	50	Median or less	Above median	24	26	0.99	0.49	1.99	0.981	46	35	
Platelet count at diagnosis	49	Median or less	Above median	25	24	1.60	0.80	3.20	0.188	44	34	
Number of chemotherapy cycles before alloHCT1	76	0-1 cycles	≥2	11	65	1.73	0.74	4.05	0.208	55	31	
Donor match at alloHCT1	76	MRD	MUD	25	36	1.55	0.84	2.87	0.162	40	24	
Donor match at alloHCT1	76	MRD	MMRD or MMUD	25	15	0.80	0.34	1.87	0.606	40	24	
Conditioning therapy alloHCT1	76	Myeloablative	Reduced intensity	23	53	1.17	0.63	2.20	0.616	46	30	
Stem cell source at alloHCT1	76	PB	BM	64	12	1.08	0.52	2.21	0.838	34	33	
Donor sex alloHCT1	76	Female	Male	33	43	1.21	0.69	2.12	0.502	39	31	
Donor age alloHCT1	75	Age 36 years or younger	Age above 36 years	38	37	0.58	0.33	1.02	0.059	21	45	5
CMV status alloHCT1	75	Patient neg. /Donor neg.	Any other constellation	26	49	1.20	0.67	2.18	0.539	42	27	
Epigenetic modifier pre DLI	76	Wildtype	Mutated	52	24	1.44	0.82	2.52	0.199	39	25	
Nucleophosmin	76	Wildtype	Mutated	72	4	0.99	0.31	3.20	0.992	35	NA	
Cohesin complex	76	Wildtype	Mutated	73	3	1.85	0.57	5.98	0.303	35	NA	

Signal transduction	76	Wildtype	Mutated	65	11	2.35	1.16	4.75	0.017	39	NA	6
Spliceosome	76	Wildtype	Mutated	67	9	1.47	0.66	3.28	0.351	36	NA	
Myeloid Transcription factor	76	Wildtype	Mutated	59	17	1.58	0.85	2.93	0.146	40	10	
Tumor suppressor	76	Wildtype	Mutated	65	11	2.29	1.13	4.63	0.021	37	18	7
DRI Score grouped	76	Low + intermediate	High + very high	47	29	1.45	0.84	2.52	0.184	38	28	
no CR vs CR and MRD negative	61	CR/CRi + MRD neg	noCR/CRi CR/CRi	23	38	5.57	2.49	12.48	<0.001	70	8	
CR and MRD+ vs CR and MRD-	38	CR/CRi + MRD neg	CR/CRi + MRD pos	23	15	3.00	1.20	7.53	0.019	70	40	
No response or CR/CRi MRD+ vs CR/CRi MRD-	76	CR/CRi + MRD-	All others	23	53	4.67	2.16	10.07	<0.001	70	17	8

Number of selected variables for multivariate analysis	8

						Overal survival						
**Variables**	**N patients**	**First category (name)**	**Second category (name)**	**First category (n)**	**Second category (n)**	**HR#**	**95% CI (lower limit)**	**95% CI (upper limit)**	**P**	**5 year NRM (%) category 1**	**5 year NRM (%) category 2**	**Selected for multivariate analysis**

pre DLI median Age	76	Age 54 years or younger	Age above 54 years	38	38	1.69	0.96	2.99	0.069	42	28	1
Patient sex	76	Female	Male	34	42	1.19	0.67	2.11	0.548	38	32	
Diagnosis	76	De novo AML	sAML / tAML / MDS/AML	44	32	1.13	0.64	1.98	0.682	36	34	
Extramedullary manifestation at diagnosis	76	No	Yes	68	8	2.89	1.28	6.56	0.011	37	NA	2
FAB subtype	51	M0 + M1 + M2	M4 + M5 + M6 + M7	29	22	1.48	0.75	2.94	0.259	38	27	
FLT3-ITD status at diagnosis	75	No	Yes	59	16	0.79	0.39	1.58	0.500	36	34	
Complex karyotype	75	No	Yes	64	11	3.10	1.50	6.40	0.002	40	NA	3
2017 ELN risk group	70	Favorable + Intermediate	Adverse	34	36	1.74	0.97	3.14	0.065	41	26	4
MRC Grimwade	75	Favorable + Intermediate	Adverse	56	19	2.35	1.27	4.36	0.007	43	11	5
ECOG performance status at diagnosis	76	ECOG 0	ECOG 1	70	6	1.66	0.60	4.64	0.332	35	33	
HCT–CI at diagnosis	76	0-2	>2	67	9	1.11	0.47	2.61	0.815	35	33	
WBC count at diagnosis	50	Median or less	Above median	24	26	1.08	0.52	2.24	0.839	41	42	
Hemoglobin at diagnosis	50	Median or less	Above median	24	26	1.15	0.55	2.39	0.708	49	37	

Platelet count at diagnosis	49	Median or less	Above median	25	24	1.36	0.66	2.83	0.404	45	36	
Number of chemotherapy cycles before alloHCT1	76	0-1 cycles	≥2	11	65	1.90	0.75	4.80	0.174	58	31	
Donor match at alloHCT1	76	MRD	MUD	25	36	1.67	0.89	3.15	0.112	37	26	6
Donor match at alloHCT1	76	MRD	MMRD or MMUD	25	15	0.70	0.29	1.72	0.436	37	26	6
Conditioning therapy alloHCT1	76	Myeloablativ e	Reduced intensity	23	53	1.30	0.68	2.49	0.434	44	31	
Stem cell source at alloHCT1	76	PB	BM	64	12	0.91	0.43	1.94	0.810	36	29	
Donor sex alloHCT1	76	Female	Male	33	43	1.03	0.58	1.83	0.909	37	33	
Donor age alloHCT1	75	Age 36 years or younger	Age above 36 years	38	37	0.56	0.32	1.00	0.050	21	45	7
CMV status alloHCT1	75	Patient neg. /Donor neg.	Any other constellation	26	49	1.18	0.64	2.17	0.594	46	27	
Epigenetic modifier pre DLI	76	Wildtype	Mutated	52	24	1.37	0.77	2.45	0.287	40	25	
Nucleophosmin	76	Wildtype	Mutated	72	4	1.23	0.38	4.00	0.725	36	NA	
Cohesin complex	76	Wildtype	Mutated	73	3	1.14	0.28	4.71	0.857	35	NA	
Signal transduction	76	Wildtype	Mutated	65	11	2.87	1.39	5.90	0.004	40	NA	8
Spliceosome	76	Wildtype	Mutated	67	9	1.11	0.47	2.62	0.806	35	33	
Myeloid Transcription factor	76	Wildtype	Mutated	59	17	1.79	0.96	3.36	0.068	41	9	9

Tumor suppressor	76	Wildtype	Mutated	65	11	2.66	1.31	5.38	0.007	38	18	10
DRI Score grouped	76	Low + intermediate	High + very high	47	29	1.60	0.91	2.83	0.103	38	30	
no CR vs CR and MRD negative	61	CR/CRi + MRD neg	noCR/CRi CR/CRi	23	38	5.67	2.45	13.1	<0.001	69	12	
CR and MRD positive vs CR and MRD negative	38	CR/CRi + MRD neg	CR/CRi + MRD pos	23	15	3.05	1.15	8.06	0.025	69	34	
No response or CR/CRi MRD+ vs CR/CRi MRD-	76	CR/CRi + MRD-	All others	23	53	4.77	2.12	10.74	<0.001	69	19	11
Number of selected variables for multivariate analysis												11

** not considered due to lack of any events

#Hazard ratios greater than or less than 1 indicate an increased or decreased risk, respectively, of an event for the second category listed.

Abbreviations: CI, confidence interval; CMV, cytomegalovirus; CR, complete remission; ECOG, Eastern Cooperative Group performance status; ELN, European LeukemiaNet; HCT, hematopoietic cell transplantation; HCT-CI, hematopoietic cell transplantation comorbidity index; HR, hazard ratio; ITD, internal tandem duplication; MMRD, mismatched related donor; MMUD, mismatched unrelated donor; MRDonor, matched related donor; MUD, matched unrelated donor; MRD, measurable residual disease; MVA, Multivariate analysis; P, P value; WBC, white blood cell count.
